# CDK2 and PKA Mediated-Sequential Phosphorylation Is Critical for p19INK4d Function in the DNA Damage Response

**DOI:** 10.1371/journal.pone.0035638

**Published:** 2012-04-25

**Authors:** Mariela C. Marazita, M. Florencia Ogara, Silvina V. Sonzogni, Marcelo Martí, Nelson J. Dusetti, Omar P. Pignataro, Eduardo T. Cánepa

**Affiliations:** 1 Laboratorio de Biología Molecular, Departamento de Química Biológica, Facultad de Ciencias Exactas y Naturales, Universidad de Buenos Aires, Ciudad de Buenos Aires, Argentina; 2 INSERM, U624 Stress Cellulaire, Marseille, France; 3 Laboratorio de Endocrinología Molecular y Transducción de señales, Instituto de Biología y Medicina Experimental-CONICET, Ciudad de Buenos Aires, Argentina; University of Pecs Medical School, Hungary

## Abstract

DNA damage triggers a phosphorylation-based signaling cascade known as the DNA damage response. p19INK4d, a member of the INK4 family of CDK4/6 inhibitors, has been reported to participate in the DNA damage response promoting DNA repair and cell survival. Here, we provide mechanistic insight into the activation mechanism of p19INK4d linked to the response to DNA damage. Results showed that p19INK4d becomes phosphorylated following UV radiation, β-amyloid peptide and cisplatin treatments. ATM-Chk2/ATR-Chk1 signaling pathways were found to be differentially involved in p19INK4d phosphorylation depending on the type of DNA damage. Two sequential phosphorylation events at serine 76 and threonine 141 were identified using p19INK4d single-point mutants in metabolic labeling assays with ^32^P-orthophosphate. CDK2 and PKA were found to participate in p19INK4d phosphorylation process and that they would mediate serine 76 and threonine 141 modifications respectively. Nuclear translocation of p19INK4d induced by DNA damage was shown to be dependent on serine 76 phosphorylation. Most importantly, both phosphorylation sites were found to be crucial for p19INK4d function in DNA repair and cell survival. In contrast, serine 76 and threonine 141 were dispensable for CDK4/6 inhibition highlighting the independence of p19INK4d functions, in agreement with our previous findings. These results constitute the first description of the activation mechanism of p19INK4d in response to genotoxic stress and demonstrate the functional relevance of this activation following DNA damage.

## Introduction

DNA damage response (DDR) mechanisms are essential for maintaining genomic integrity and an accurate transmission of genetic information. DDR consists of an intricate signaling network in which complex DNA surveillance programs play a key role [1]–[3]. These control programs or checkpoints respond to a variety of lesions including stalled replication forks and DNA damage induced by both internal and external sources like reactive cellular metabolites, ionizing or UV radiation and chemotherapeutic agents [2], [4], [5]. After sensing the damage, the activation of the checkpoints modulate cell cycle arrest, DNA repair systems and cell death mechanisms to repair or to eliminate hazardous, genetically unstable cells [6], [7]. Although DDR components have not yet been completely described the canonical checkpoint signaling is composed by two major transduction pathways initiated by the upstream PI3K-like kinases Ataxia-telangiectasia Mutated (ATM) and ATM and Rad3-related (ATR). ATM is predominantly activated by double strand break lesions (DSBs) while ATR responds fundamentally to single strand breaks or bulky lesions. ATM and ATR activate their downstream kinases Chk1 and Chk2 amplifying the initial signal and modulating the G_1_/S, intra-S and G_2_/M checkpoints [4], [8]. While ATM and ATR were initially reported to activate Chk2 and Chk1 respectively, this concept was challenged by studies that show crosstalks between these kinases [9]. Chk1 activation by ATM was reported in cells exposed to ionizing radiation treatment [10], [11] and ATM and ATR were required for Chk2 activation in response to replication stress [12]. Moreover, it was shown that both ATR and ATM were able to target the SQ-rich C terminus of Chk1 on serine 317 and 345 leading to its activation [10], [13]–[15]. Following Chk1 and Chk2 activation, these kinases phosphorylate a wide range of downstream effectors which prevent further progression through the cell cycle and initiate DNA repair mechanisms but also modulate the trigger of cell death pathways if the insult exceeds the repair capacity [2], [16]. Among these effector proteins, Chk1 phosphorylates TLK12 and RAD51, while BRCA, PIK3, PML and E2F1 are Chk2 substrates. They also share target proteins like Mdm2, p53, cdc25A and cdc25C [5], [17]–[20]

The cell cycle progression is driven by the activity of cyclin-dependent kinases (CDKs) and is negatively regulated by INK4 and Cip/Kip inhibitory proteins [21]–[24]. INK4 family consists of four members, p16INK4a, p15INK4b, p18INK4c and p19INK4d which play a redundant role as CDK4/6 inhibitors. However, novel cell cycle independent functions were recently described for some of them [25]. Interestingly, p16INK4a and p19INK4d (p19) were linked to the cellular response to genotoxic agents [26]–[28]. In particular, extensive data points out that p19 is a critical factor in the maintenance of genomic integrity and cell survival. It was reported that UV light, cisplatin and β-amyloid peptide promoted p19INK4d transcriptional induction and nuclear translocation [27]. Adding to this, p19 overexpression significantly enhanced DNA repair and diminished apoptosis in different cell lines. More important, physiological p19 levels are necessary for an appropriate response to the damage. In this way, p19 deficient cells display an impaired DNA repair activity and enhanced apoptosis [27]–[29]. Consistent with these findings, other studies described enhanced sensitivity of cells to apoptosis and autophagic cell death in p19 null mice [30]. p19 expression status directly correlates with cell resistance and survival to DNA damage. Finally, p19 activity protects from UV-induced chromosomal aberrations and spontaneous chromosome abnormalities as well [27], [29]. These facts uncovered a novel p19 function in regulating genomic stability and overall cell viability under conditions of genotoxic stress.

Despite these findings, the regulation of p19 activity in the DDR remains unknown. We hypothesized that post-translational modifications on p19 could be taking part in regulating this specific function. Here, we report the activation mechanism of p19 in response to DNA damage. It is shown that this event is dependent on the ATM/ATR signaling pathways and occurs in a sequential manner at serine 76 (S76) and threonine 141 (T141). These two identified phosphorylation sites would be direct substrates for CDK2 and PKA kinases respectively. Moreover, DNA damage induced p19 nuclear translocation requires S76 phosphorylation. And finally, both phosphorylation sites are shown to be crucial for p19 function in DNA repair and cell survival but dispensable for CDK4/6 inhibition. These results position p19 in a novel context as a downstream target of the DDR signaling pathways, provide mechanistic insight into the activation mechanism of p19INK4d in response to DNA damage and demonstrate the functional relevance of this activation following genotoxic stress.

## Materials and Methods

### Cell culture, transfections and antibodies

WI-38 cell line was grown in minimum essential medium supplemented with 10% fetal bovine serum (FBS) and 1% penicillin-streptomycin, 100 mM non-essential aminoacids, and 2 mM glutamine at 37°C in a humidified 5% CO2 atmosphere. HEK-293 cells were grown in Dulbecco's modified Eagle medium supplemented as described above. Transfections were performed using Lipofectamine 2000 Reagent (Invitrogen). Approximately, 2×10^6^ cells were seeded in 60-mm plates and transfected with 4 µg of wild type or mutant p19 expression plasmids. 24 h after transfection, cells were treated as indicated in each experiment. For UDS, [^3^H]thymidine incorporation and caspase-3 activity assays, WI-38 cells were plated in 35-mm dishes at a density of 1×10^6^ cells/well in 2.5 ml of medium. After a 24 h attachment period, each well was transfected with a mixture containing 2 µg of wild type or mutant p19 expression plasmids and 0.5 µg of pBabePuro per plate. Twenty-four hours after transfection, 2.5 µg/ml puromycin were added for 48 h to select for transfected cells.

Antibodies: p19 (P1000-38, USBiological), p19 (sc-1063, Santa Cruz Biotechnology, INC), V5 (R960-25, Invitrogen), V5 (sc-83849-R, Santa Cruz Biotechnology, INC). CDK2 (sc-163, Santa Cruz Biotechnology, INC), PKAc(C-20, Santa Cruz Biotechnology), histone H3 (sc-8654–R, Santa Cruz Biotechnology). GAPDH (AB8245, ABCAM). Anti-mouse and anti-rabbit secondary antibodies conjugated with horseradish peroxidase were purchased from SIGMA (Saint Louis, USA).

### UV irradiation

Cells were irradiated in open dishes with UV (4 mJ/cm^2^), 254 nm (range 240–280 nm) at room temperature using a Philips ultraviolet lamp (TUV15WG15T8) calibrated to deliver 0.25 mJ/cm^2^ s. Following UV irradiation, medium was replaced and cells were further incubated for the indicated times. For each experiment, control cells were treated identically except for UV light exposure.

### Metabolic labeling of p19 in WI-38 cells with sodium orthophosphate - ^32^P

WI-38 cells were grown in 60-mm dishes and treated as indicated. Before treatment, cells were incubated with 0,5 mCi sodium orthophosphate-^32^P for 3 h. At indicated time points, cells were washed, collected in cold PBS and lysed in RIPA buffer. The lysates (100 µg) were incubated with the appropiate antibody for 2 h, followed by O.N. incubation with protein AG agarose beads at 4°C. After washing three times with RIPA buffer, samples were analyzed by immunoblotting or SDS-PAGE. Dried gels were exposed to a radiographic intensifying screen by Fujifilm and scanned directly using a Bio-Imaging Analyzer Fujifilm BAS-1800II.

### Plasmids

Construction of p19 mutants is described in Supporting Information (Materials and Methods S1).

### Downregulation of CDK1 and CDK2

Antisense oligonucleotides (AS) complementary to either human CDK1 or CDK2 (corresponding to bases +129 to +155 and +46 to +73 respectively) were transfected with Lipofectamine 2000. At a final concentration of the AS was 1 µM. After 12 h, the medium was replaced by fresh medium containing 1 µM of the corresponding AS. Cells were treated for *in vivo* phosphorylation as described previously. ASCDK1: 5′ tattttggtattatcttccatagttag 3′; ASCDK2: 5′ccaacttgaaacaatcttgccgcctccc 3′.

### p19 structure and Molecular Dynamics Simulations

Analysis of p19 structure was performed using the VMD programmed (Visual Molecular Dynamics, http://www.ks.uiuc.edu/Research/vmd/). Simulations of p19 phosphorylations were obtained using AMBER software. XY graphs were done using XMGRACE utility. (CA positions, alpha carbon positions).

### Analysis of potential phosphorylation sites and kinase-specific prediction of phosphorylation sites

Netphos 2.0 server [31], a neural network-based method for predicting potential phosphorylation sites at serine, threonine or tyrosine residues, was used to analyze p19 protein sequence (http://www.cbs.dtu.dk/services/NetPhos/). NetphosK 1.0 server was used for kinase-specific prediction of phosphorylation sites [32] (http://www.cbs.dtu.dk/services/NetPhosK/).

### Alignment of protein sequences

Protein sequences were aligned using T-Coffee multiple sequence alignment tool [33].

### RNA extraction and Northern blot analysis

Total cellular RNA was isolated from cultures as described previously [34]. Ten micrograms of total RNA were denatured, electrophoresed in 1% glyoxal/agarose gels, and transferred to nylon membranes (GeneScreen Plus, PerkinElmer). The membranes were sequentially hybridized with ^32^P-labeled probes to CDK1, CDK2 and β-ubulin. To detect CDK1 mRNA, a 28-mer ODN was synthesized complementary to bases +124 to +151 of human p19 mRNA. To detect CDK2 mRNA, a 29-mer ODN was synthesized complementary to bases +45 to +73 of human p19 mRNA. To detect β-tubulin a 22-mer ODN was synthesized complementary to bases +174 to +195 of human tubulin beta 3 mRNA. ODN were 5-end-labeled using [γ-^32^P**]** ATP and T4 polynucleotide kinase. Hybridization was carried as previously described [27]. Membranes were exposed to a radiographic intensifying screen by Fujifilm and scanned directly using a Bio-Imaging Analyzer Fujifilm BAS-1800II.

### CDK2 kinase assay

CDK2 kinase assay was performed as described by Giono et. al. [35]. Briefly, HEK 293 cells were washed with PBS and lysed in HB buffer Proteins (1 mg) were immunoprecipitated with 10 µl of anti-CDK2 antibody and 50 µl of a 50% slurry of protein A/G-agarose beads (SIGMA) and rocked at 4°C for 2 h. For control sample containing only the substrate (GST-p19) but no enzyme, proteins were incubated with the beads without anti-CDK2 antibody. The beads were washed and resuspended in 15 µl of HB buffer. To each sample was added 15 µl of 2× kinase reaction buffer (HB buffer containing 200 µM ATP, 1 mg/ml GST-p19, and 1 µl of [γ-^32^P]ATP (6,000 Ci/mmol [10 µCi/µl]) per 10 µl of 2× reaction buffer). Samples were incubated for 20 min at 37°C. The reaction was stopped by addition of 7.5 µl of 5× sample buffer and 5 min incubation at 95°C. Histone H1 from calf thymus (Calbiochem) was used for kinase activity control. Samples were electrophoresed on 12% denaturing gels. Gels were dried on Whatman paper, exposed to a radiographic intensifying screen by Fujifilm and scanned directly using a Bio-Imaging Analyzer Fujifilm BAS-1800II. In a similar assay, phosphorylation of a p19 derived peptide containing the surrounding sequence of S76 (p-S76; RGTSPVHDAART; 200 mM) was tested. As control for CDK2 activity, an histone H1 derived peptide (p-H1; PKTPKKAKKL). Following the reaction, samples were processed according to the phosphocellulose paper method. As a negative control, the reaction was conducted without substrate.

### PKA kinase assay

The *in vitro* phosphorylation assays were performed in a final volume of 40 µl of reaction buffer (50 mM potassium phosphate, pH 7.5, 0.1 mM EGTA, 0.1 mM EDTA, 15 mM MgCl_2_, 10 mM 2-mercaptoethanol, 0.1 mM [γ-^32^P]ATP (700 dpm/pmol) 0,1 mM ATP, 100 µg/ml PMSF, 60 µg/ml aprotinin, 1 mM sodium orthovanadate) plus 5 µg of GST-p19 with or without H-89 (100 µM). After 15 min at 30°C, samples were electrophoresed on 12% denaturing gels. Gels were dried on Whatman paper, exposed to a radiographic intensifying screen by Fujifilm and scanned directly using a Bio-Imaging Analyzer Fujifilm BAS-1800II. In a similar assay, phosphorylation of a p19 derived peptide containing the surrounding sequence of threonine 141 (p-T141; RDARGLTPLELA; 200 mM) was tested. As control for PKA activity, Kemptide was used as substrate (kemp; LRRASLG). Following the reaction, samples were processed according to the phosphocellulose paper method. As a negative control, the reaction was conducted without substrate.

### PKA-p19 co-immunoprecipitation

Co-immunoprecipitation assays were performed by transfection of pcDNA4cp19wt in WI-38 cells. A total of 500 µg of proteins were immunoprecipitated with 3 µl of anti-V5 antibody and 30 µl of 50% slurry of protein A/G agarose beads (SIGMA). The beads were washed three times with RIPA, resuspended in 30 µl of 2× sample buffer, and heated to 95°C for 5 min. Proteins were resolved in 15% polyacrylamide gels and analyzed by immunoblotting.

### Caspase-3 activity

Cells were treated with UV (4 mJ/cm^2^) or 10 µM β-amyloid peptide for 12 h. Cells were then harvested with lysis buffer (50 mM Tris–HCl, pH 7.4, 1 mM EDTA, 10 mM EGTA, 10 µM digitonine, 0.5 mM PMSF, 10 µg/ml pepstatin, and 10 µg/ml aprotinin) incubated for 30 min at 37°C and centrifuged at 12,000× *g* for 20 min. The activity of caspase-3 in 150 µl of cell lysate was determined using 100 µM of the synthetic caspase-3 substrate Ac-DEVD-pNA (Sigma) in reaction buffer (100 mM HEPES pH 7.5, 0.5 mM EDTA, 5 9 mM dithiothreitol and glicerol 20% v/v) in a final volume of 300 µl and incubated at 37°C during 4 h. Color development was measured at 405 nm. Caspase activity was estimated as *A*405/µg protein.

### Subcellular fractionation

Cells were seeded on 100 mm-plates and transfected with the indicated p19 expression plasmids. After 24 h, 1 µM H-89 was added for 1 hour treatment. Cells were treated with 4 mJ/cm^2^ UV and harvested at the indicated times. Subcellular fractionation was performed as described by Schreiber *et al* [36] with minor modifications. Briefly, cells were washed with 10 ml PBS, collected in 1 ml PBS and collected by centrifugation at 1500× g for 5 min. Cell pellets were resuspended in 400 µl of cold buffer A (10 mM HEPES pH 7.9; 10 mM KCl; 0.1 mM EDTA; 0.1 mM EGTA; 1 mM DTT; 0.5 mM PMSF) and cells were allowed to swell on ice for 15 min. Twenty five µl of a 10% solution of Nonidet NP-40 were added and the tube was vigorously vortexed for 10 sec. Homogenates were centrifuged for 30 sec in a microcentrifuge. Supernatants (cytoplasmic fraction) were transferred to a fresh tube and pellets (nuclear fraction) were resuspended in 90 µ1 ice-cold buffer C (20 mM HEPES pH 7.9; 0.4 M NaCl; 1 mM EDTA; 1 mM EGTA; 1 mM DTT; 1 mM PMSF) and the tube was vigorously rocked at 4°C for 15 min on a shaking platform. Nuclear extracts were centrifuged for 5 min and supernatants transferred to a clean tube. Cytoplasmic and nuclear extracts were analyzed for *in vivo* p19 or p19-V5 phosphorylation as mentioned before.

### [^3^H]thymidine incorporation

Twenty four hours after transfection, cells were incubated with 1 µCi/ml [^3^H]-thymidine (81 Ci/mmol) (Amersham Biosciences) for 6 h. Cells were washed three times with cold PBS, harvested, and centrifuged at 3000×g for 5 min. The cellular pellet was lysed with 5% trichloroacetic acid (TCA) for 30 min, centrifuged and washed twice with cold H_2_O_2_. The pellet was resuspended in 150 µl 1 M NaOH for 1 h at room temperature. Incorporated radioactivity was quantified by scintillation counting and DNA synthesis expressed as dpm/µg protein.

### Unscheduled DNA synthesis

Twenty four hours after transfection, cells were washed with PBS and growth medium was replaced by arginine-free medium containing 1% FBS which was renewed after 24 h. Inhibition of DNA semiconservative synthesis was confirmed under these conditions. Cells were treated with UV (4 mJ/cm^2^), or 10 µM β-amyloid peptide and further cultured in UDS medium and 10 µCi/ml [^3^H]thymidine. At the indicated times, cells were washed three times with cold PBS, harvested and collected at 3000×*g* for 5 min. Cells were lysed with 5% TCA for 30 min. and centrifuged at 10,000×*g* for 10 min. Pellet was washed twice with cold PBS and resuspended in 1 M NaOH. The incorporated radioactivity was quantified by scintillation counting. Unscheduled DNA synthesis was expressed as dpm/µg protein.

## Results

### p19INK4d phosphorylation in response to DNA damage

**Figure 1:**
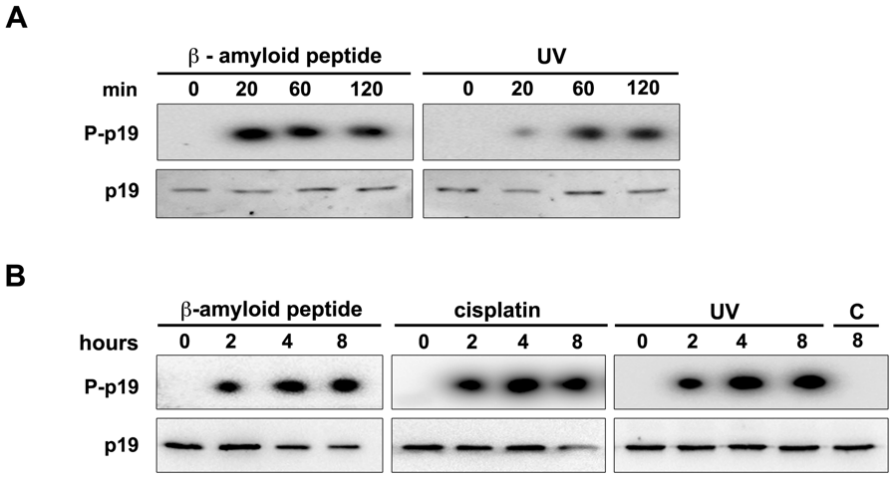
p19 phosphorylation is induced in response to DNA damage. (**A, B**) WI-38 fibroblasts were labeled with [^32^P]-orthophosphate and treated with β-amyloid peptide (20 µM), cisplatin (10 µM) or UV light (4 mJ/cm^2^) for the indicated times. Equal amounts of whole cell extracts were subjected to immunoprecipitation with anti-p19 antibody and the immune complexes were analyzed by SDS-PAGE and autoradiography (upper panels; P-p19, phosphorylated p19) or immunoblotting (lower panels; p19). (C; Control, untreated cells).

We have previously reported that p19 is involved in DNA repair, genome maintenance and cell survival [27]–[29]. Then, we aimed to study the mechanism by which p19 is activated in response to DNA insults. It was hypothesized that p19 could be target of the phosphorylation pathways activated by DNA damage. To test this, p19 phosphorylation status was examined after treatment with three different genotoxic agents: UV light, cisplatin and β-amyloid peptide. *In vivo* phosphorylation analyses were performed by metabolic labeling of WI-38 fibroblasts. Under basal conditions, no phosphorylation of endogenous p19 was observed. In contrast, p19 rapidly became phosphorylated 20 minutes after β-amyloid treatment or UV exposure (Figure 1A). The phosphorylation signal remained elevated for at least 8 hours after treatment with all three damaging agents (Figure 1B, Figure S1). These results show that p19 becomes phosphorylated following DNA damage.

### p19INK4d is sequentially phosphorylated in serine 76 and threonine 141 upon DNA damage

To further study p19 phosphorylation, the protein sequence was analyzed for the presence of potential phosphorylation residues (Figure S2). Five p19 mutants were constructed replacing serine or threonine by alanine at the predicted phosphorylation sites and the phosphorylation capacity of these mutants was assessed *in vivo* by metabolic labeling.

**Figure 2:**
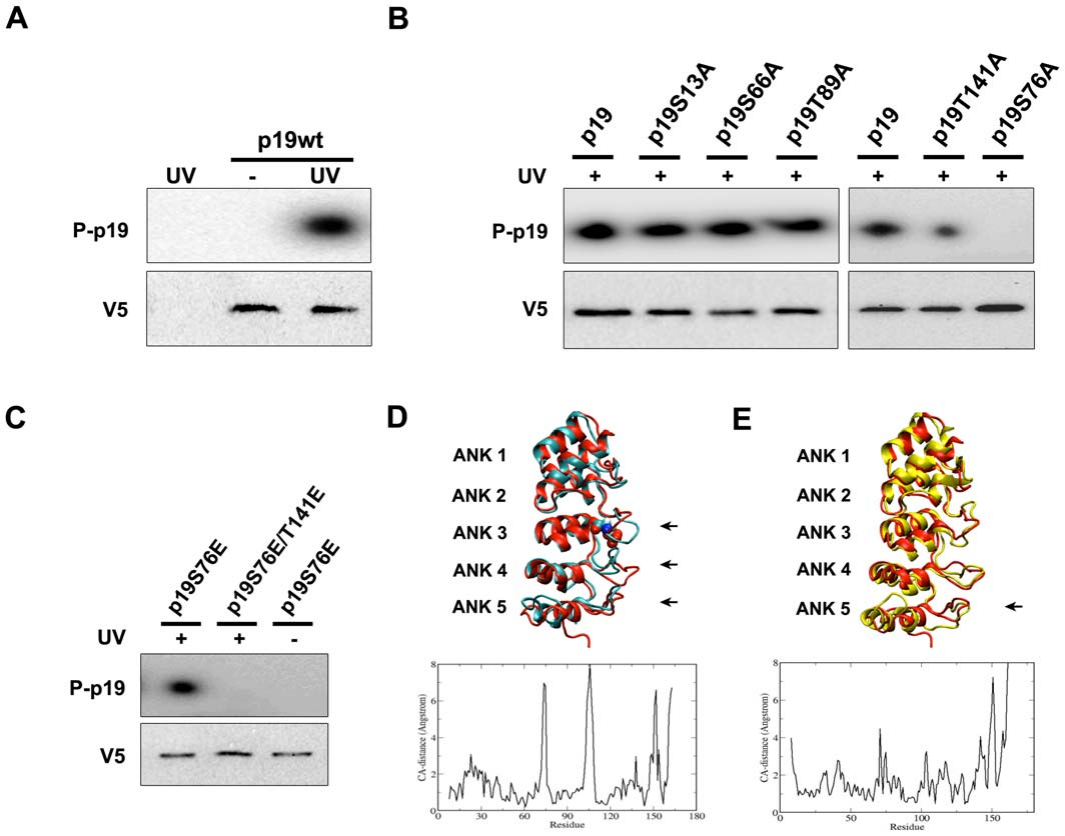
Sequential phosphorylation of p19 at S76 and T141 following DNA damage. (**A, B**) Phosphorylation ability of p19 mutants. WI-38 fibroblasts were transfected with expression vectors encoding the V5 epitope tag in frame with wild type p19 (p19wt) or p19 mutants, in which the potential phosphorylation sites were replaced by alanine (p19S13A, p19S66A, p19S76A, p19T89A, p19T141A). Transfected cells were labeled with [^32^P]-orthophosphate, treated with UV light (4 mJ/cm^2^) and collected 3 hours after treatment. Extracts were subjected to immunoprecipitation with anti-V5 antibody and analyzed by autoradiography (upper panels, P-p19) or immunoblotting (lower panels, V5). Unstransfected cells were used as a control to monitor immunoprecipitation specificity. (**C**) In a similar experiment, *in vivo* phosphorylation of two p19 phosphomimetic mutants (p19S76E and p19S76E/T141E) was tested. (**D, E**) Structural changes promoted by the sequential phosphorylation were analyzed by molecular dynamics simulation. The images show the comparison between the structures of p19 (cyan) and p19 phosphorylated on S76 (p19p, red) (**D**), or between p19p and p19 phosphorylated on sites S76 and T141 (p19pp, yellow) (**E**). Graphs show CA-distance between p19 and p19p (**D**), or p19p and p19pp (**E**) average structures. Arrows indicate domains with predicted structural changes. (ANK 1–5, ankyrin domains 1 to 5).

Phosphorylation of overexpressed p19 was absent in untreated cells and was induced after UV radiation (Figure 2A). p19S13A, p19S66A and p19T89A mutants showed phosphorylation levels comparable to p19wt. In contrast, p19T141A and p19S76A displayed relevant differences. While p19T141A phosphorylation was significantly reduced, phosphorylation of p19S76A was completely abolished (Figure 2B). These results strongly suggested that S76 and T141 were actual target sites for phosphorylation *in vivo*. In addition, the lack of phosphorylation on p19S76A raised the hypothesis of a two-step process in which the modification of T141 would be dependent on the phosphorylation of S76. To study this possibility, two glutamic acid mutants were generated mimicking the phosphorylation effect at S76 (p19S76E) or at both sites, S76 and T141 (p19S76E/T141E). In accordance with the hypothesis of a sequential phosphorylation, the phosphomimetic mutation at S76 enabled the phosphorylation of p19S76E mutant at T141 (Figure 2C). Interestingly, no p19S76E phosphorylation was observed in the absence of UV irradiation. Then, an active DNA damage response pathway is required to undergo a second modification at a site different from S76. Moreover, no phosphorylation was detected in p19S76E/T141E after genotoxic treatment. These results are in agreement with those showing decreased and lack of signal in p19T141A and p19S76A respectively and hence support S76 and T141 as the only phosphorylation residues.

The potential effects of the phosphorylation on p19 structure were analyzed by Molecular Dynamics Simulation. p19 is composed of five ankyrin repeats of about 30–35 residues long. Each repeat consists of a β-hairpin followed by two anti parallel α-helices. S76 and T141 are located in the third and fifth ankyrin domain respectively, at the end of the β-hairpin. When phosphorylation at S76 was simulated (p19p) direct comparison between p19 and p19p average structures showed significant differences (Figure 2D). Up to 8 Å between the CA positions were observed for key structural regions. The main structural changes were found in the β-hairpins of the third ankyrin repeat, where the phosphoserine is positioned, and also in the fourth repeat. In p19 structure both loops are close together but the presence of the phosphate pushed them away. An increase in mobility was also found in the loop between helices I and II of the fifth repeat in which T141 is situated. Supporting p19 sequential phosphorylation, the structural changes induced by S76 modification could be necessary for the interaction between p19 and the second kinase responsible for T141 phosphorylation. When both sites were phosphorylated (p19pp) the main differences compared to p19p were located in the fifth repeat (Figure 2E). The change in the β-hairpin loop of the repeat was smaller than the change observed for the loop between both helices. The presence of the phosphate broke the R135-E144 interaction. E144 was pushed away and a strong interaction between R135 and the phosphate group was established. These additional changes resulted particularly interesting since they might promote the interaction between p19 and proteins related to its function in the DDR.

Overall, we propose a sequential phosphorylation model for p19 in which modification at S76 would enable a second phosphorylation event at T141. The phosphorylation-induced structural changes could have functional implicancies for p19 in the DNA damage response.

### ATM-Chk2 and ATR-Chk1 checkpoint pathways are differentially involved in p19 phosphorylation

**Figure 3:**
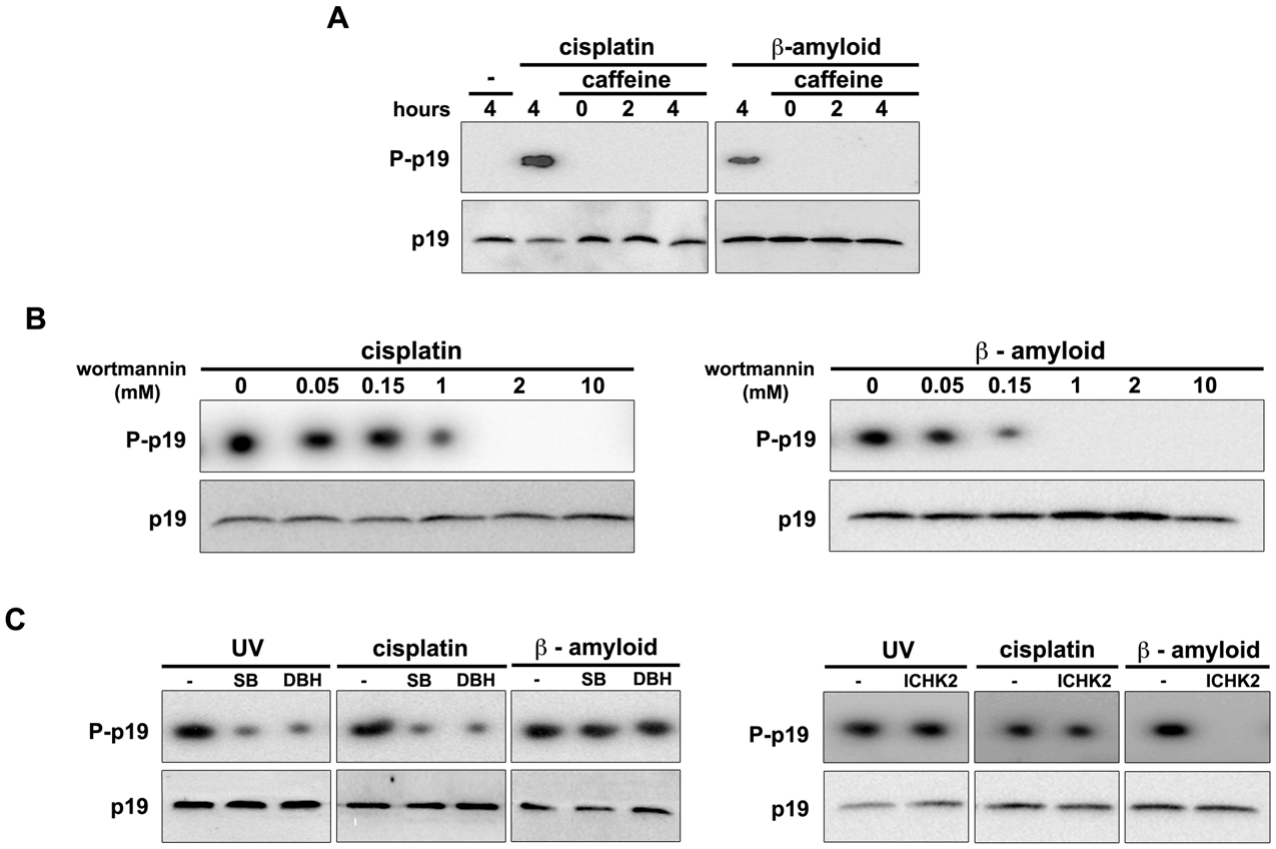
ATM/ATR signaling pathways are differentially involved in p19 phosphorylation. (**A**) Inhibition of p19 phosphorylation by caffeine treatment. WI-38 fibroblasts were incubated with caffeine (5 mM) for 1 hour, then treated with cisplatin (10 µM) or β-amyloid peptide (20 µM) for the indicated times and endogenous p19 phosphorylation analyzed by autoradiography. (**B**) Evaluation of ATM/ATR involvement in p19 phosphorylation by wortmannin treatment. WI-38 fibroblasts were incubated with the indicated doses of wortmannin for 1 hour, followed by treatment with cisplatin (10 µM) or β-amyloid peptide (20 µM) for 2 hours. (**C**) Effect of Chk1 and Chk2 inhibitors on p19 phosphorylation. WI-38 fibroblasts were incubated with SB-218078 (SB, 15 nM) or dopamine ß-hidroxylase inhibitor (DBH, 3 µM), both Chk1 inhibitors, or with Chk2 Inhibitor Calbiochem (ICHK2, 20 nM) for 1 hour before treatment with UV light (4 mJ/cm^2^), cisplatin (10 µM) or β-amyloid peptide (20 µM). After 2 hours, cell extracts were analyzed as in A.

ATM and ATR kinases are members of the PIKKs family that play a major role in the DDR. It was previously reported that UV radiation and cisplatin cause DNA damage which promotes predominantly ATR activation, while β-amyloid peptide mainly stimulates ATM activity [37], [38] **.** Since we demonstrated that p19 phosphorylation is induced by all three DNA damaging agents, we hypothesized that the ATM/ATR pathway could be involved in this process. To examine this possibility, caffeine, a specific PIKKs inhibitor, was used in the analysis of p19 phosphorylation *in vivo*. Caffeine treatment effectively prevented p19 phosphorylation (Figure 3A). Additionally, wortmannin, a PIKKs and PI3K inhibitor, was used to conduct a similar analysis. It was reported that different wortmannin concentrations are needed to inhibit the diverse kinases (PI3K and DNA-PKc IC50: 0.016 µM; ATM IC50: 0.15 µM; ATR IC50: 1.8 µM) [39]. A dose-response curve of wortmannin was performed to assess its inhibitory effect on p19 phosphorylation. As expected, the concentration of wortmannin required to block p19 phosphorylation depended on the genotoxic agent tested. The phosphorylation promoted by β-amyloid peptide was diminished by 0.15 µM wortmannin and totally suppressed at the concentration of 1 µM, doses reported to inhibit ATM but not ATR (Figure 3B, right panel). In contrast, cisplatin-induced p19 phosphorylation was completely abolished only at high wortmannin concentrations (2 µM) necessary to inhibit ATR (Figure 3B, left panel). Therefore, these results suggested that both ATM and ATR signaling pathways promote p19 phosphorylation and that they act in response to different types of DNA damage.

Chk1 and Chk2 kinases amplify the signals initiated by ATM/ATR. Then, *in vivo* p19 phosphorylation was examined after treatment with Chk1 or Chk2 inhibitors. Results showed that p19 phosphorylation promoted by UV light or cisplatin was impaired by Chk1 inhibition (Figure 3C, left panel). In contrast, Chk2 inhibitor suppressed p19 phosphorylation only when the damage was induced by β-amyloid peptide. These results are consistent with the fact that Chk1 and Chk2 are predominantly activated by ATR and ATM respectively and further support the data presented in figure 3B.

We conclude that there is a differential involvement of ATM-Chk2 and ATR-Chk1 pathways in p19 phosphorylation which depends on the type of lesion in the DNA.

### p19 phosphorylation requires CDK and PKA activities

**Figure 4:**
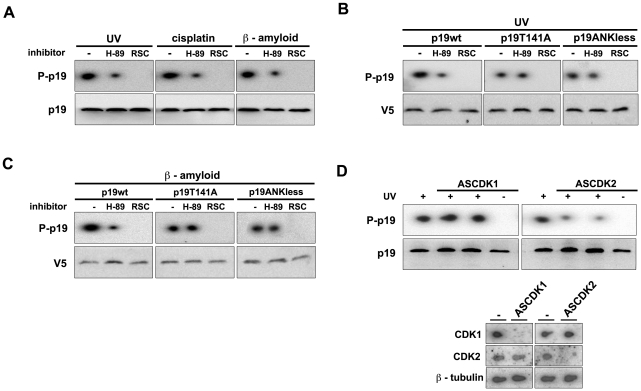
CDK2 and PKA participate in p19 sequential phosphorylation. (**A**) CDK and PKA involvement in endogenous p19 phosphorylation. WI-38 fibroblasts were incubated with roscovitine (RSC, 10 µM), or with H-89 (1 µM) for 1 hour before the damaging treatments (4 mJ/cm^2^ UV light, 10 µM cisplatin or 20 µM ß-amyloid peptide). p19 phosphorylation was analyzed by autoradiography. (**B, C**) Effect of CDK and PKA inhibition on the phosphorylation of T141 mutants. WI-38 cells were transfected with the indicated p19 constructs expression plasmids, incubated with roscovitine or H-89 for 1 hour and then treated with UV light (4 mJ/cm^2^) or β-amyloid peptide (20 µM) for 2 hours. p19wt or the mutants were immunoprecipitated with anti-V5 antibody and the immunocomplexes were analyzed by autorradiography and immunoblotting. (**D**) Measurement of CDK1 and CDK2 activities in the phosphorylation process of endogenous p19. WI-38 fibroblasts were incubated for 24 hours with specific CDK1 or CDK2 antisense oligonucleotides before treatment with UV radiation (4 mJ/cm^2^). After 2 hours, p19 was immunoprecipitated and phosphorylation observed by autoradiography as mentioned before (upper panel). Northern blot results show the efficiency of the antisense oligonucleotides (lower panel).

ATM-Chk2 and ATR-Chk1 activates numerous phosphorylation pathways in response to DNA insults leading to the repair of the damage or ultimately to cell death. We aimed to investigate which pathways and particularly which kinases were directly involved in p19 phosphorylation. As an initial approach, a search for potential kinases predicted CDK5 and PKA acting at S76 and T141 respectively (Figure S3). CDK5 is a serine/threonine kinase with high sequence homology to CDK1 and CDK2 [40]–[42]. The brain is the only tissue that shows CDK5 histone H1 kinase activity and no equivalent kinase activity has been found in other tissue culture cell lines [43]. The substrate specificity of CDK1 and CDK2 is similar to that of CDK5 phosphorylating the (S/T)PX(K/H/R) consensus sequence motif [44], [45]. In p19, S76 corresponds to a perfect consensus site constituted by the sequence SPVH. To evaluate the involvement of these enzymes, specific kinase inhibitors were used in phosphorylation assays *in vivo*. H-89 treatment, a specific inhibitor of PKA, partially decreased endogenous p19 phosphorylation induced by UV radiation, β-amyloid peptide and cisplatin treatment (Figure 4A). A concentration of H-89 20 times higher than the one used in figure 4A and reported to abolish PKA activity in various cell types was unable to further diminish the phosphorylation (Figure S4). Interestingly, the decrease in p19 phosphorylation after PKA inhibition was similar to that observed for p19T141A (Figure 2B). This fact is consistent with the *in silico* analysis which predicted PKA as the kinase acting on T141. Adding to this, roscovitine, a potent inhibitor of CDK1, CDK2 and CDK5 kinases, completely blocked p19 phosphorylation induced by the three DNA damaging treatments tested, supporting the prediction of the CDK activity on S76 (Figure 4A). Moreover, PKA inhibition did not affect neither p19T141A nor p19ANKless (a p19 mutant lacking the last ankyrin repeat where T141 is positioned) phosphorylation with the genotoxic drugs tested (Figure 4B–C). These results suggest that there is no other site different from threonine 141 where PKA activity might be involved. Furthermore, roscovitine treatment completely blocked the phosphorylation of both mutants, p19T141A and p19ANKless (Figure 4B–C). Since only two residues become phosphorylated after DNA injury, these observations indicate that S76 should be the specific target site for the CDK activity. These results also support the hypothesis of a sequential phosphorylation which would be dependent on CDK and PKA activities.

We next aimed to identify the CDK family member necessary for p19 phosphorylation acting in this process. Since CDK5 activity was only reported in neural cells, the involvement of CDK1 and CDK2 kinases was examined in the cell lines tested. Specific antisense oligonucleotides were used to down-regulate either CDK1 (ASCDK1) or CDK2 (ASCDK2). The efficiency and specificity of the antisense oligonucleotides was first tested by Northern blot (Figure 4D, lower panels**).** *In vivo* p19 phosphorylation was not affected by ASCDK1 treatment. In contrast, a decrease in the phosphorylation was found following ASCDK2 treatment suggesting a role for CDK2 in this process.

In summary, these observations are consistent with the sequential phosphorylation of p19 involving CDK2 function on S76 that would enable the activity of PKA on T141.

### CDK2 and PKA phosphorylates p19 *in vitro*

**Figure 5:**
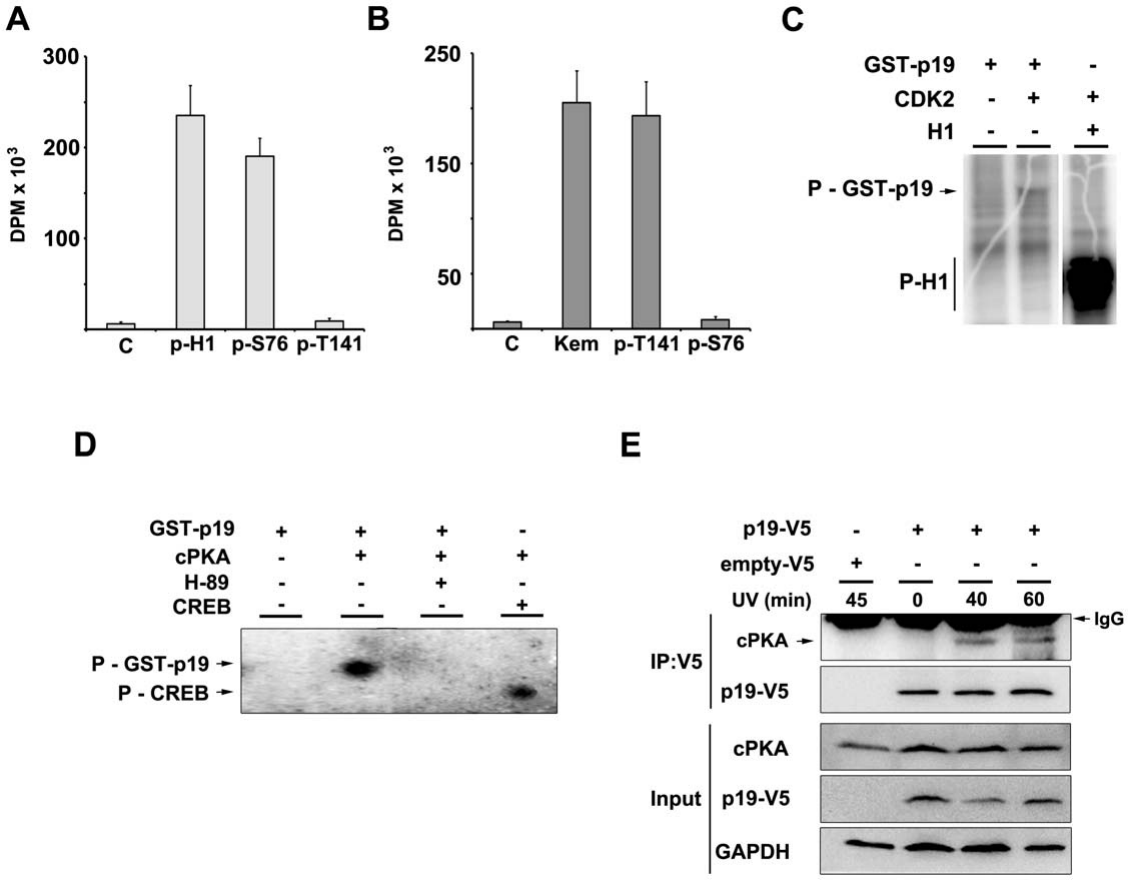
CDK2 and PKA phosphorylates p19 *in vitro.* (**A, B**) S76 and T141 as suitable sites for CDK2 and PKA action. Two synthetic peptides containing the sequence in which S76 (p-S76) or T141 (p-T141) are positioned, were used to performed *in vitro* kinase assays. p-S76 or p-T141 peptides were incubated with CDK2 (immunoprecipitated from HEK-293 cells) or the catalytic subunit of PKA (cPKA, purified from bovine heart), respectively. A histone H1 peptide (p-H1) or kemptide (Kemp) were used as specific subtrates for CDK2 and PKA, respectively, as a control of enzymatic activity. Kinase activity specificity was tested by substituting one substrate to the other. Measurements were done in triplicates and bars show the mean ± s.e.m. (n = 3). (**C**) CDK2 phosphorylates p19. *In vitro* kinase assays were performed using immunoprecipitated CDK2 and recombinant GST-p19. Histone H1 was used as a control for CDK2 activity. (**D**) PKA phosphorylates p19. *In vitro* kinase assays were performed using cPKA and recombinant GST-p19 as substrate, with or without H-89 inhibitor. CREB protein was used as a control for cPKA activity (**E**) Analysis of the interaction between PKA and p19 *in vivo*. Co-immunoprecipitation assays were performed transfecting p19-V5 (p19wt) in WI-38 cells. Cells were irradiated with UV light. At the indicated times following irradiation treatment cells were collected and the extracts immunoprecipitated with anti-V5 antibody (IP:V5). The immune complexes were analyzed by immunoblot with anti-cPKA and anti-V5 antibodies. Expression of p19-V5 and cPKA was analyzed in the inputs by immunoblot.

We have demonstrated that CDK2 and PKA activities are required for p19 phosphorylation. To investigate whether p19 is a direct target of CDK2 and PKA we carried out *in vitro* kinase assays using specific p19 derived peptides or recombinant GST-p19 protein as substrates. In the first approach, two synthetic peptides containing either the sequence of S76 (p-S76: RGTSPVHDAART) or T141 (p-T141: RDARGLTPLELA) phosphorylation sites were employed to test the direct activity of CDK2 or PKA correspondingly. Results showed that CDK2 was able to efficiently phosphorylate the p-S76 peptide (Figure 5A). PKA catalytic subunit (cPKA) was capable of phosphorylating the p-T141 peptide as observed by ^32^P incorporation (Figure 5B). Taken together, the data indicates that S76 and T141 are contained within a suitable consensus phosphorylation site for CDK2 and PKA respectively and support the direct action on p19.

It was examined whether CDK2 and PKA could phosphorylate p19 *in vitro* using bacterially expressed and purified GST-p19. *In vitro* phosphorylation assays were performed incubating GST-p19 either with bovine heart-purified cPKA or CDK2 immunoprecipitated from HEK 293 cells. Results showed phosphorylated p19 when CDK2 activity was tested (Figure 5C). In a similar analysis, GST-p19 was also phosphorylated by cPKA (Figure 5D). These findings indicate that p19 is a proper substrate for the activity of both kinases CDK2 and PKA.

The ability of PKA to interact with p19 was investigated by co-immunoprecipitation assays. After transfection of p19wt and following UV irradiation, immunoprecipitated p19wt was found associated to cPKA, confirming the interaction *in vivo* (Figure 5E). Probably because of the weak and fast kinase-substrate association, p19 interaction with CDK2 could not be observed.

Taken together, results from both *in vitro* and *in vivo* phosphorylation assays support the direct phosphorylation of p19 by CDK2 and PKA.

### Serine 76 phosphorylation regulates p19 nuclear translocation

It was previously reported that p19 translocates from the cytoplasm to the nucleus following genotoxic insult [27]. However, p19 protein sequence does not reveal a nuclear localization signal. We thus hypothesized that the phosphorylation might promote the relocalization of p19.

**Figure 6:**
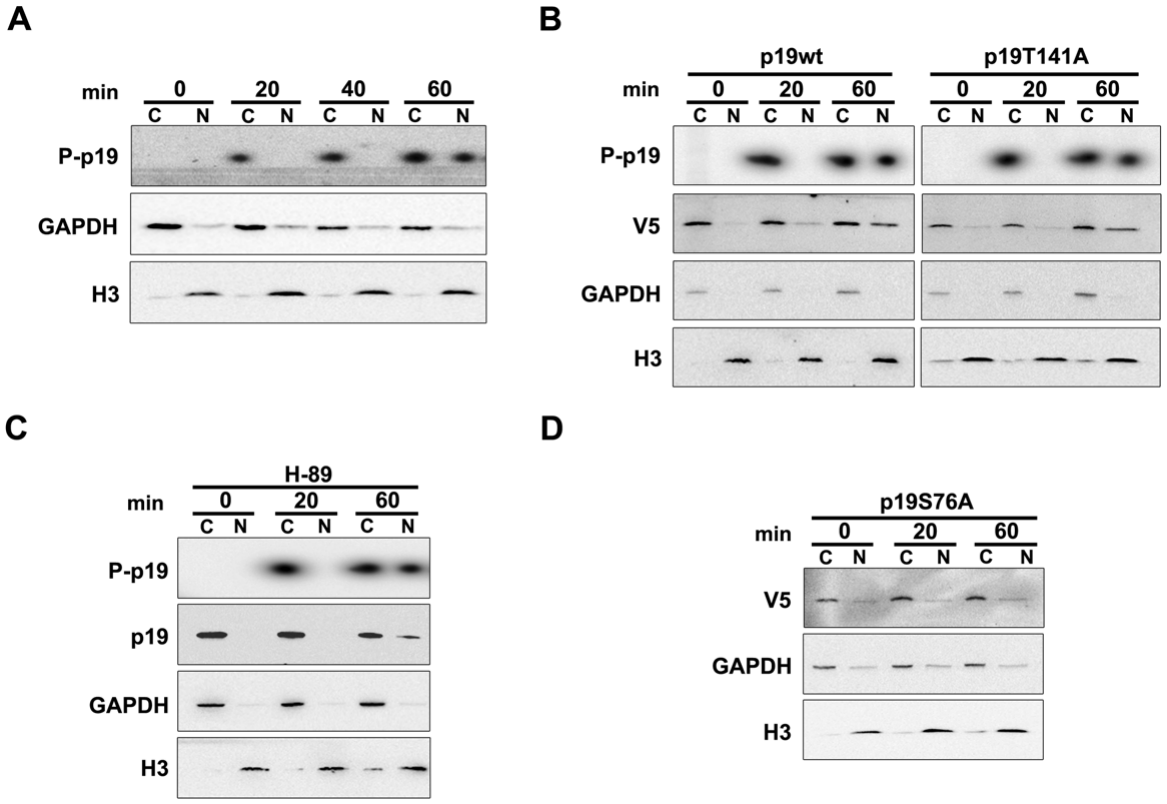
DNA damage induced p19 nuclear translocation is dependent on S76 phosphorylation. (**A**) Distribution of phosphorylated p19 in the cytoplasmic and nuclear fractions after DNA damage. *In vivo* phosphorylation assays were performed in WI-38 fibroblasts. Cells were treated with UV (4 mJ/cm2), collected at the indicated times, and the extracts subjected to a subcellular fractionation protocol. Either the cytoplasmic (C) or nuclear fractions (N) were immunoprecipitated with anti-p19 antibody, and the immunocomplexes analyzed by SDS-PAGE and autoradiography (upper panel). (**B**) Subcellular distribution of the phosphorylation deficient mutant p19T141A. For *in vivo* phosphorylation assays, WI-38 cells were transfected with p19wt or p19T141A, treated with UV radiation and collected at the indicated times. After subcellular fractionation, extracts were immunoprecipitated with an anti-V5 antibody and analyzed as in (**A**). p19wt or p19T141A subcellular distributions were also studied by immunoblot (**C**) Subcellular localization of endogenous deficiently phosphorylated-p19 after PKA inhibition. For *in vivo* phosphorylation assays, cells were processed as in (**A**) but, before UV irradiation, they were incubated with H-89 for 1 hour. Endogenous distribution of p19 was also studied by immunoblot. (**D**) Subcellular localization of p19S76A mutant following DNA damage. WI-38 cells were transfected with p19S76A and treated with UV radiation. At the indicated times, extracts were prepared by subcellular fractionation and analized by immunoblot with anti V5-antibody.

We first aimed to determine the subcellular compartment in which p19 phosphorylation occurred. *In vivo* phosphorylation assays were performed adding a subcellular fractionation step before the immunoprecipitation of endogenous p19 or p19wt. Phosphorylated p19 showed a cytoplasmic localization at 20 and 40 minutes following the damage, whereas at 60 minutes p19 appeared in the nuclear fraction (Figure 6A). The intracellular distribution of phosphorylation deficient mutants showed that p19T141A conserved the ability to translocate into the nucleus (Figure 6B). A similar result was observed for endogenous p19 when PKA was inhibited by H-89 (Figure 6C). The analyses of protein distribution patterns by western blot were consistent with the phosphorylation results. In contrast p19S76A, the mutant completely lacking phosphorylation, lost the nuclear import induced by DNA damage (Figure 6D).

As a whole, these results indicate that T141 phosphorylation is dispensable for p19 nuclear translocation while S76 phosphorylation would be crucial in this process.

### Serine 76 and threonine 141 phosphorylation is critical for p19 function linked to the response to DNA damage

We next examined the functional relevance of p19 phosphorylation. As previously mentioned, p19 is a cell cycle inhibitor which has also a role in the DDR. Then, the ability to inhibit cell cycle progression was first assessed for p19 mutants. The results showed that all of the mutants (p19S13A, p19S66A, p19S76A, p19T89A, p19T141A, p19S76A/T141A) displayed similar abilities to block cell proliferation compared to p19wt (Figure S5). Therefore, neither S76 nor T141 are necessary for inhibiting CDK4/6.

**Figure 7:**
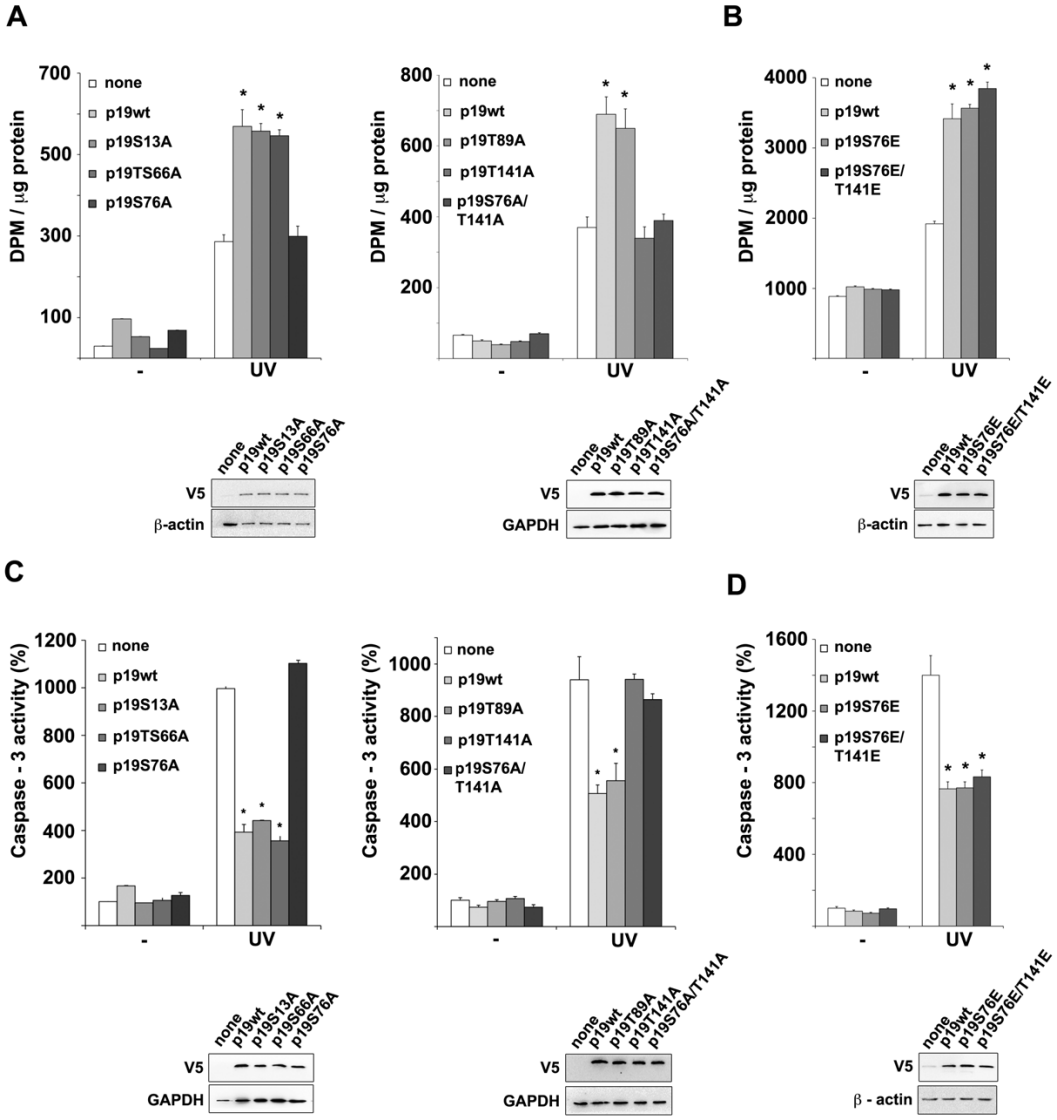
Phosphorylation of serine 76 and threonine 141 is required for p19 function linked to the response to DNA damage (**A**) DNA repair ability of cells overexpressing p19wt or p19 phosphorylation deficient mutants. WI-38 fibroblasts were transfected with p19wt or the indicated p19 mutants. Cells were maintained in an arginine-free medium containing 1% fetal bovine serum during 48 h, damage with 4 mJ/cm^2^ UV and incubated with [^3^H]-thymidine. Following 10 h, cell lysates were tested for Unscheduled DNA Synthesis assay (UDS). Bars represent the mean ± s.e.m of three independent experiments performed in triplicate. Student's *t*-test was used to compare UV-treated control sample (none) with UV-treated p19wt or p19 mutant samples. (*p<0,005). Protein expression was analyzed by immunoblot. (**B**) Similarly as in (**A**) but overexpressing the phosphomimetic p19 mutants. (**C**) UV-dependent apoptotic response of cells overexpressing p19wt or phosphorylation deficient mutants of p19. WI-38 fibroblasts were transfected with p19wt or the indicated p19 mutants. Twelve hours following UV irradiation, cell lysates were tested for caspase-3 activity. Results are expressed as percentage of caspase-3 activity with respect to basal activity of cell lysates nontransfected and without UV-treatment, which was set to 100. Bars represent the mean ± s.e.m. of three independent experiments performed in triplicate. Student *t*-test was used to compare, UV-treated control sample (none) with UV-treated p19wt or p19 mutant samples (^*^ *p*<0.005). (**D**) Similarly as in (**C)** but overexpressing the phosphomimetic p19 mutants.

The DDR involves complex signal transduction pathways that regulate DNA repair and cell death mechanisms to restore DNA integrity or to eliminate the damaged cell. We have previously reported that p19 participates in the DDR being necessary for an efficient repair of the DNA damage [25], [27]–[29]. Particularly, down regulation of endogenous p19 resulted in decreased DNA repair after treatment with different genotoxic drugs. In contrast, p19 overexpression showed enhanced DNA repair activity compared to non transfected cells. To study the functional role of p19 phosphorylation, the DNA repair ability of the cells overexpressing p19 mutants was analyzed by Unscheduled DNA Synthesis (UDS). The overexpression of those which maintain a complete phosphorylation capability (p19S13A, p19S66A or p19T89A) achieved similar levels of DNA repair compared to those observed for p19wt (Figure 7A and Figure S6A). However, when any of the phosphorylation deficient-mutants were tested (p19S76A, p19T141A, p19S76A/T141A), DNA repair levels were significantly diminished after UV light or β-amyloid treatment, reaching those values obtained for control cells (Figure 7A and Figure S6A). In contrast, the glutamic acid mutants mimicking the phosphorylation at S76 and T141 (p19S76E, p19S76E/T141E) recovered the DNA repair function displaying levels comparable to p19wt (Figure 7B and Figure S6B). These results show that the phosphorylation on both sites, S76 and T141, are strictly necessary for p19 role in DNA repair. Since S76 and T141 are dispensable for the inhibition of the cell cycle, these findings also support that the role of p19 as a cell cycle regulator is dissociated from its DNA repair function (27).

Two mechanisms are essential in response to genotoxic stress to maintain genome integrity: DNA repair and apoptosis. As part of the DDR, when the DNA damage is too severe to be repaired, cell death programs are activated to eliminate the cell irreversibly affected. It was previously reported that p19 overexpression significantly decreases the apoptosis induced by UV light, cisplatin and β-amyloid peptide [27], [28]. Then, we tested whether p19 phosphorylation mutants, lacking the DNA repair activity, also lost the ability to protect cells against apoptosis. All the mutants with full phosphorylation capability reduced caspase-3 activity to a level similar to that observed for p19wt. Conversely, cells overexpressing the phosphorylation-deficient mutants displayed higher levels of caspase-3 activity that were comparable to those measured in control cells (Figure 7C and Figure S7). In contrast, the mutants mimicking the phosphorylation on S76 and T141 (p19S76E, p19S76E/T141E) showed values similar to those found for p19wt (Figure 7D). We then concluded that those mutations which disrupt p19 phosphorylation also affect the protection conferred by p19 from apoptosis.

Altogether, these findings are consistent with a regulation pathway in which S76 and T141 phosphorylation is critical for the reported function of p19 linked to the response to DNA damage.

## Discussion

In response to DNA damage, conserved checkpoint mechanisms trigger multiple events that coordinate cell cycle progression, DNA repair and cell death in order to restore DNA integrity or to eliminate the irreversibly damaged cell. The present study uncovers the activation mechanism of p19 in response to DNA damage. The results show that p19 is a downstream target of the main DDR signaling pathways, ATM/Chk2 and ATR/Chk1. It was demonstrated that p19 function involved in DNA repair and cell survival is modulated by a sequential phosphorylation at serine 76 and threonine 141 dependent on CDK2 and PKA. A previous work reported p19 phosphorylation under basal conditions in U2OS cell line and suggested serines 66 and 76 as phosphorylation sites [46]. Although in our study p19 phosphorylation was not observed without genotoxic treatment in any of the cell lines tested, the previous findings are consistent with S76 as a potential phosphorylation aminoacid. Remarkably, both identified residues, S76 and T141, are conserved in p19 protein sequences of different mammal species but not throughout the other INK4 family members (Figure S8, S9). Then, the phosphorylation and activation mechanism presented herein might explain the singular function in the DDR of this INK4 protein.

The structural and dynamic consequences caused by the phosphorylations on p19 were analyzed by Molecular Dynamics (MD). Simulation of S76 phosphorylation (p19p) showed a main increase in the mobility of the third ankyrin motif, where the negative charge was located, but also mobility differences in the fourth and fifth ankyrin repeat were observed. The experimental data indicated a sequential phosphorylation at S76 and T141 aminoacids. Supporting the sequential phosphorylation results, the structural changes in the fifth ankyrin repeat induced in p19p might be necessary to enable T141 phosphorylation. The main differences found when both, S76 and T141, phosphorylations were simulated are located in the fifth repeat. We propose that the additional changes in the structure promoted by the second phosphorylation could be involved in the interaction of p19 with proteins related to DDR mechanisms and then might be critical for p19 function. Further experimental studies are being conducted to identify p19 interactors and verify this possibility.

It is well established that inhibition of CDK activity, one of the main actions promoted by checkpoint responses, leads to cell cycle arrest and provides time for DNA repair. However, increasing evidence supports an active role for CDKs in the DDR. Overall CDK activity was reported to be necessary for an efficient DDR activation after γ-irradiation induced DNA damage [47]. In yeasts and mammalian cells, CDK activity is essential for DNA resection and progression of homologous recombination repair during S and G2 phases [19], [48]–[50]. For instance, CDK2 targets several substrates in the DDR pathway such us BRCA1 and BRCA2, Ku70 and ATRIP [51], [52]. Cyclin A1 which promotes CDK2 activation is transcriptionally induced by γ and UV-irradiation via a p53-mediated mechanism [51]. Interestingly, this fact contrasts with the inhibition and/or repression of the other CDK2-associated cyclins. Consistent with these reports, the sequence analysis of S76 matched an exact CDK2 phosphorylation motif, suggesting that p19 might be a putative substrate for this kinase. *In vitro* assays confirmed direct CDK2-mediated phosphorylation of p19. Adding to this, CDK inhibition prevented DNA-damage-induced phosphorylation of endogenous p19. Specific down-regulation of CDK2 impaired p19 phosphorylation. Together, these results show the dependence of p19 phosphorylation on CDK2 function and strongly suggest the direct action of this kinase on this protein. *In vivo* CDK inhibition also blocked the phosphorylation of p19T141A and p19ANKless mutants. Since only two residues, S76 and T141, become phosphorylated after DNA injury these observations also indicate that S76 might be the specific target site for CDK2. T141 in p19 was shown to be embedded within a PKA consensus motif. PKA has been shown to exert an antiapoptotic effect in different cell lines. In addition, PKA activity was implicated in the activation of the processivity factor PCNA and in the nuclear translocation of DNA-PK, two critical proteins in DNA repair [52]–[54]. Herein, phosphorylation and interaction assays performed *in vitro* and *in vivo* supported the direct action of PKA on p19. Moreover, the decreased phosphorylation observed for endogenous p19 after H-89 treatment was consistent with a reduced phosphorylation of p19T141A and p19ANKless mutants. Even more, no further reduction in p19T141A or p19ANKless phosphorylation was found by PKA inhibition, suggesting that the action site of this kinase had already been eliminated in these mutants. Taken together, these findings support p19 phosphorylation by PKA in response to DNA damage and point out to T141 as the target site for this kinase.

The regulation of protein localization provides cells with a convenient way to modulate their functions. p19 does not contain the standard basic monopartite or bipartite nuclear localization signal usually found in nuclear proteins [55]. However, protein phosphorylation also serves as an essential mechanism for modulating subcellular localization. To analyze this possibility, the cellular compartment in which p19 phosphorylation takes place was explored. Phosphorylation assays and immunoblot analysis showed phosphorylated p19 in the cytoplasm followed by a translocation into the nucleus. Moreover, p19T141A was also able to translocate into the nucleus in spite of its phosphorylation deficiency. In contrast, p19S76A lost the nuclear import induced by DNA damage. Consequently, these results suggest that the first phosphorylation event on serine 76 would allow p19 nuclear translocation while modification of T141 would be dispensable in this matter. In view of the results discussed before, these findings imply the presence of active CDK2-cyclin A complexes in the cytoplasm. During cell cycle progression, the activity of CDKs is located in the nucleus. However, consistent with our findings recent works showed cytoplasmic translocation of active CDK2 in response to UV irradiation and chemotherapeutic agents [56]. In addition, cytoplasmic CDK2 activity was related to apoptotic cell death [57]. There is accumulating evidence supporting the fact that some proteins involved in DNA repair may also be taking part in apoptosis. [25], [58]. Thus, CDK2 might also be among these proteins playing a dual role in the DDR, modulating the activity of both anti apoptotic and pro-apototic proteins. Since p19 nuclear translocation was only dependent on S76, it is tempting to speculate that the phosphorylation on T141 might occur in the nucleus. In addition to the structural changes promoted by S76 phosphorylation, the nuclear import preceding T141 phosphorylation further supports the sequential phosphorylation of p19.

Protein phosphorylation is a widely used mechanism to selectively modulate protein activity. We then investigated if phosphorylation had a functional relevance on p19. The expression of p19 mutants lacking S76 and/or T141 promoted cell cycle arrest at similar levels to those observed for wild type p19. These results indicate that neither S76 nor T141 are necessary for p19 inhibition of CDK4/6 kinases. Previous works based on crystal structure analysis showed that binding to CDK6 involves mainly ankyrin domains I–III of p19. In accordance with our findings, threonine 141 is positioned within the fifth ankyrin repeat and then would not participate in the interaction with CDK. Moreover, S76, located in the third ankyrin repeat, was not described to be implicated in CDK binding by NMR studies. [59]–[61]. In contrast, both S76 and T141 phosphorylation were found to be crucial for p19 function related to the response to DNA damage. Since the phosphorylation-deficient mutants keep the ability to block cell cycle progression, the results suggest that p19 activity linked to the DDR is not associated with inhibiting cell proliferation. In fact, these findings denote the independence between the functions of p19 in the cell cycle and in the DDR, in agreement with our previous works [27], [29].

In summary, our results uncover the activation mechanism of p19 implicated in the response to DNA damage. We propose that the phosphorylation of specific sites might induce conformational changes in p19 necessary for the correct subcellular localization and for the interaction with DDR proteins. Mutations in DDR critical genes that lead to impaired genome stability, increased cancer susceptibility or enhanced cell death reflect the importance of a proper DDR. Consequently, a comprehensive knowledge of the DDR pathways becomes essential to understand disease development and might contribute to establish more efficient therapeutic approaches.

## Supporting Information

Materials and Methods S1 **Description of the mutagenesis strategy used to construct p19 mutants.**
(DOC)

Figure S1 **p19 immunoprecipitation specificity.** WI-38 fibroblasts were labeled with [^32^P]-orthophosphate and treated with β-amyloid peptide (20 µM), cisplatin (10 µM) or UV light (4 mJ/cm^2^) for 3 hours. Equal amounts of whole cell extracts were subjected to immunoprecipitation with anti-p19 antibody (+, rabbit IgG, Santa Cruz Biotechnology) or anti-V5 antibody as a control antibody (−, rabbit IgG, Santa Cruz Biotechnology). The immune complexes were analyzed by SDS-PAGE and autoradiography (upper panels; P-p19, phosphorylated p19) or immunoblotting (lower panels; p19).
(TIF)

Figure S2 **Prediction of p19 phosphorylation sites.** p19 protein sequence was analyzed for the presence of potential phosphorylation sites using the bioinformatic tool Netphos 2.0 server. Tables show serine predictions (**A**) or threonine predictions (**B**), no putative tyrosine phoshorylation sites were found. (**C**) Graph shows the score of the predicted phosphorylation sites. Pos, position of the potential phosphorylation site.
(TIF)

Figure S3 **Prediction of kinase specific phosphorylation sites in p19.** p19 protein sequence was analyzed for the presence of kinase specific phosphorylation sites using the bioinformatic tool NetphosK 1.0 server with evolutionary stable sites filter (ESS filter). Table shows the position of the putative phosphorylation sites for the indicated kinases. (Pos, position in p19 protein sequence).
(TIF)

Figure S4 **p19 phosphorylation is not abolished by high concentrations of PKA inhibitor.** WI-38 fibroblasts were incubated with the indicated concentrations of H-89 for 1 hour, and then treated with cisplatin (10 µM) for 2 hours and endogenous p19 phosphorylation analyzed by autoradiography.
(TIF)

Figure S5 **S76 and T141 are not involved in the cell cycle function of p19.** Proliferation status of cells overexpressing p19wt or p19 phosphorylation deficient mutants. WI-38 fibroblasts were transfected with p19wt or the indicated p19 mutants. Cells were incubated with [^3^H]-thymidine for 5 hours and the lysates were tested for tritium incorporation. Bars represent the mean ± s.e.m of three independent experiments performed in triplicate. Student's *t*-test was used to compare control sample (none) with p19wt or p19 mutant samples. (*p<0,005).
(TIF)

Figure S6 **Phosphorylation of S76 and T141 is required for p19 function in DNA repair.** (**A**) DNA repair ability of cells overexpressing p19wt or p19 phosphorylation deficient mutants. WI-38 fibroblasts were transfected with p19wt or the indicated p19 mutants. Cells were maintained in an arginine-free medium containing 1% fetal bovine serum during 48 h. β-amyloid peptide (20 µM) was added to the medium and cells were incubated with [^3^H]-thymidine for 10 hours. Cell lysates were tested for Unscheduled DNA Synthesis assay (UDS). Bars represent the mean ± s.e.m of three independent experiments performed in triplicate. Student's *t*-test was used to compare β-amyloid peptide-treated control sample (none) with β-amyloid peptide-treated p19wt or p19 mutant samples. (*p<0,005). Protein expression was analyzed by immunoblot. (**B**) Similarly as in (**A**) but overexpressing the phosphomimetic p19 mutants.
(TIF)

Figure S7 **Phosphorylation of S76 and T141 is necessary for p19 function in apoptosis.** β-amyloid peptide-dependent apoptotic response of cells overexpressing p19wt or the phosphorylation deficient mutants, p19S76A and p19T141A. WI-38 fibroblasts were transfected with p19wt or the indicated p19 mutants. β-amyloid peptide (20 µM) was added to the medium and following 12 hours cell lysates were tested for caspase-3 activity. Results are expressed as percentage of caspase-3 activity with respect to basal activity of cell lysates nontransfected and without β-amyloid peptide-treatment, which was set to 100. Bars represent the mean ± s.e.m of three independent experiments performed in triplicate. Students *t*-test was used to compare, β-amyloid peptide-treated control sample (none) with β-amyloid peptide-treated p19wt or p19 mutant samples (^*^ *p*<0.005).
(TIF)

Figure S8 **Conservation of p19 phosphorylation sites in different mammalian species.** p19 protein sequences from the indicated mammals were align using T-Coffee multiple sequence alignment tool. Arrows indicate the position of S76 and T141 from p19 human sequence.
(TIF)

Figure S9 **Alignment of protein sequences of the INK4 family members.** Protein sequences were align using T-Coffee multiple sequence alignment tool. Arrows indicate the position of S76 and T141 from p19 protein sequence. (p15, p15INK4b; p16, p16INK4a; p18, p18INK4c; p19, p19INK4d).
(TIF)
