# Supplementary material for: CDK2 and PKA Mediated-Sequential Phosphorylation Is Critical for p19INK4d Function in the DNA Damage Response
Source: PLoS One. 2012 Apr 25;7(4):e35638. doi: 10.1371/journal.pone.0035638 (PMC3338453; doi:10.1371/journal.pone.0035638)
Supplement: Materials and Methods S1 — Description of the mutagenesis strategy used to construct p19 mutants. (DOC) [file pone.0035638.s001.doc]

**Materials and Methods S1**

**Plasmids**

The cDNA sequence of human p19 was subcloned into pcDNA^TM^4/V5-HisC (BaHMI –EcoRI, p19wt) by PCR using the following primers: 5´cgggatcccgatgctgctggaggaggttcgc 3´ (forward), 5´ggaattcctcccagcggggccaccatgtg 3´(reverse). Mutagenesis at serine 13 and threonine 141 were generated by PCR using p19wt template and the following primers: p19S13A 5´cgggatcccgatgctgctggaggaggttcgcgccggcgacccggctggccggggcg3´ (forward), 5´ggaattcctcccagcggggccaccatgtg3´ (reverse); p19T141A 5´cgggatcccgatgctgctggaggaggttcgc3´ (forward), 5´ggaattcctcccagcggggccaccatgtggccctgcaggatgtccacgaggtcctgagcccctctctgca gtgccagctccaagggggcgagacc 3´ (reverse); p19ANKless, p19 mutant lacking the fifth ankyrin repeat, 5´cgggatcccgatgctgctggaggaggttcgc3´ (forward), 5´ ggaattcccctggcgtccctgcgatggaga 3´(reverse); p19S76A/T141A, p19S76A mutant was used as template for this PCR reaction, 5´cgggatcccgatgctgctggaggaggttcgc3´(forward), 5´ggaattcctcccagcggggccaccatgtggccctgcaggatgtccacgaggtcctgagcccctctctgcagtgccagctccaagggggcgagacc 3´(reverse); p19S76E/T141E was generated using the plasmid bearing the mutation S76E as template, 5´cgggatcccgatgctgctggaggaggttcgc 3´ (forward), 5´ggaattcctcccagcggggccaccatgtggccctgcaggatgtccacgaggtcctgagcccctctctgcagtgcc agctccaagggttcgagacc 3´ (reverse). Other mutations were performed following the “mismatched primer mutagenesis” protocol (Higuchi, 1990). Briefly , Mutations are introduced at a specific point within a chosen sequence using mismatched [primers](http://www.ncbi.nlm.nih.gov/books/n/hmg/A3037/def-item/A3298/). Two mutagenic reactions are designed in which the two separate PCR products have partially overlapping sequences containing the mutation. The denatured products are combined to generate a larger product with the mutation in a more central location. To generate the two separate PCR products the following primers were used: for p19S66A mutant 5´gtcctggacattgggggcggcaccttgctcag3´ (reverse), 5´ctgaagcaaggtgccgcccccaatgtccaggac3´ (forward); p19S76A 5´ctgcgtcatggactggagcggtacccggagg 3´(reverse), 5´gacacctccggtaccgctccagtccatgacgcag 3´ (forward); for p19T89A mutant 5´cactaggaccttcagggcgtccaggaatccagt 3´ (reverse) 5´actggattcctgga cgccctgaaggtcctagtg 3´ (forward); for p19S76E mutant 5´ ctgcgtcatggactggttcggtaccggaggtgtc 3´ (reverse), 5´ gacacctccggtaccgaaccagtccatgacgcag 3´ (forward). All these PCR mutant fragment were subcloned into pcDNA4^TM/^V5-HisC with the same primers used for p19wt.
