# Supplementary figures and images for: CDK2 and PKA Mediated-Sequential Phosphorylation Is Critical for p19INK4d Function in the DNA Damage Response
Source: PLoS One. 2012 Apr 25;7(4):e35638. doi: 10.1371/journal.pone.0035638 (PMC3338453; doi:10.1371/journal.pone.0035638)

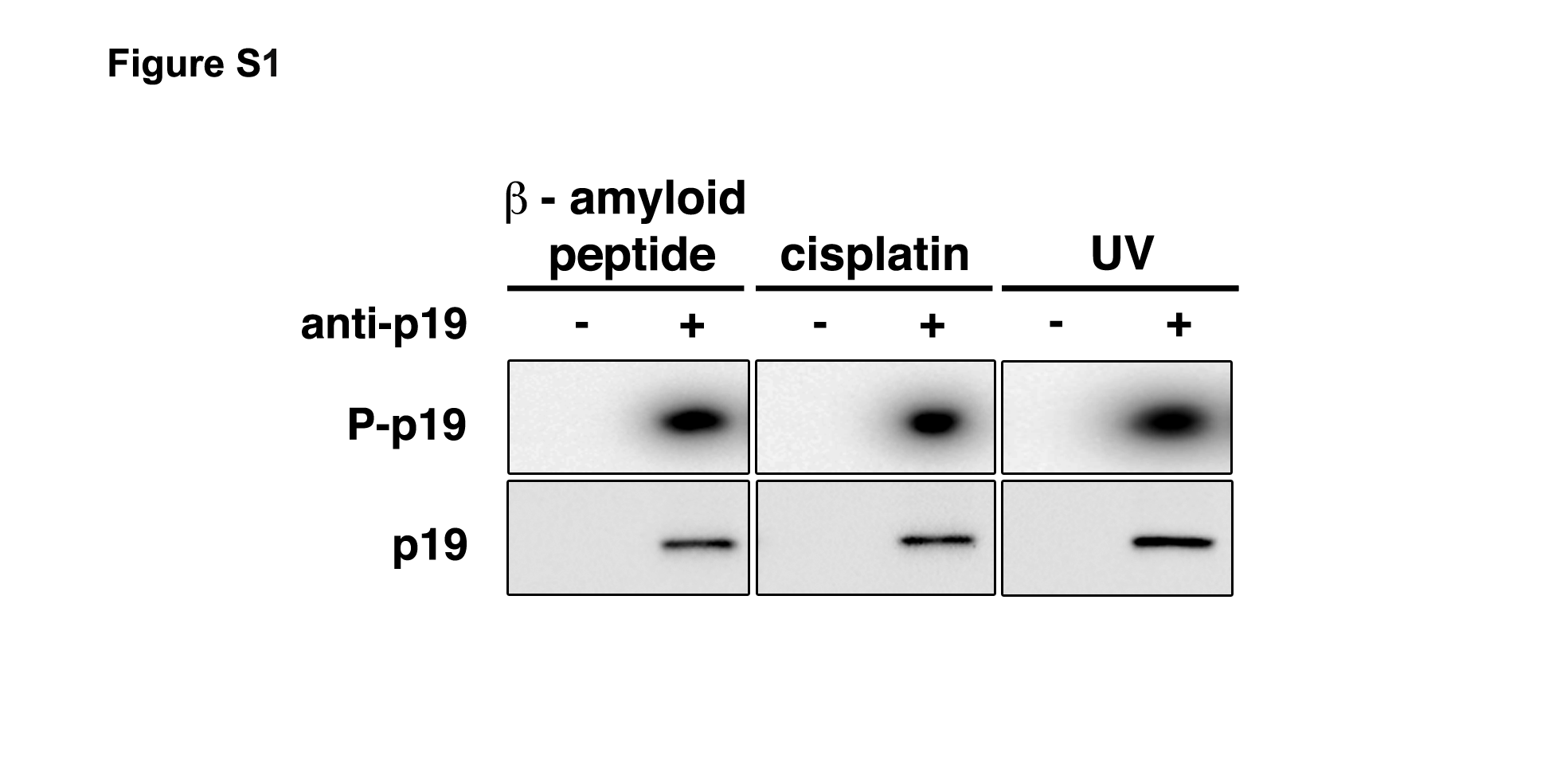

Supplement: Figure S1 — p19 immunoprecipitation specificity. WI-38 fibroblasts were labeled with [32P]-orthophosphate and treated with β-amyloid peptide (20 µM), cisplatin (10 µM) or UV light (4 mJ/cm2) for 3 hours. Equal amounts of whole cell extracts were subjected to immunoprecipitation with anti-p19 antibody (+, rabbit IgG, Santa Cruz Biotechnology) or anti-V5 antibody as a control antibody (−, rabbit IgG, Santa Cruz Biotechnology). The immune complexes were analyzed by SDS-PAGE and autoradiography (upper panels; P-p19, phosphorylated p19) or immunoblotting (lower panels; p19). (TIF) [file pone.0035638.s002.tif]

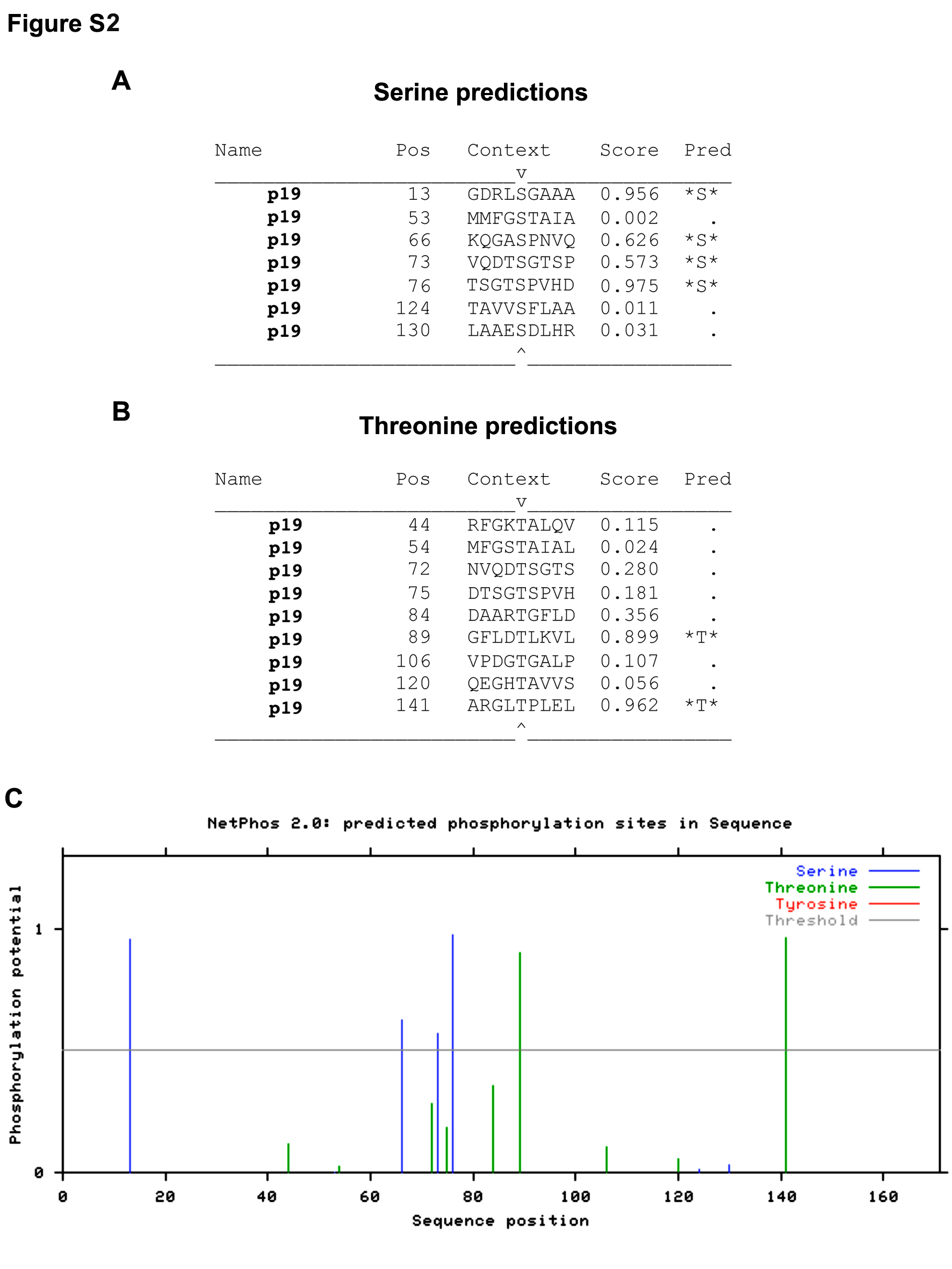

Supplement: Figure S2 — Prediction of p19 phosphorylation sites. p19 protein sequence was analyzed for the presence of potential phosphorylation sites using the bioinformatic tool Netphos 2.0 server. Tables show serine predictions (A) or threonine predictions (B), no putative tyrosine phoshorylation sites were found. (C) Graph shows the score of the predicted phosphorylation sites. Pos, position of the potential phosphorylation site. (TIF) [file pone.0035638.s003.tif]

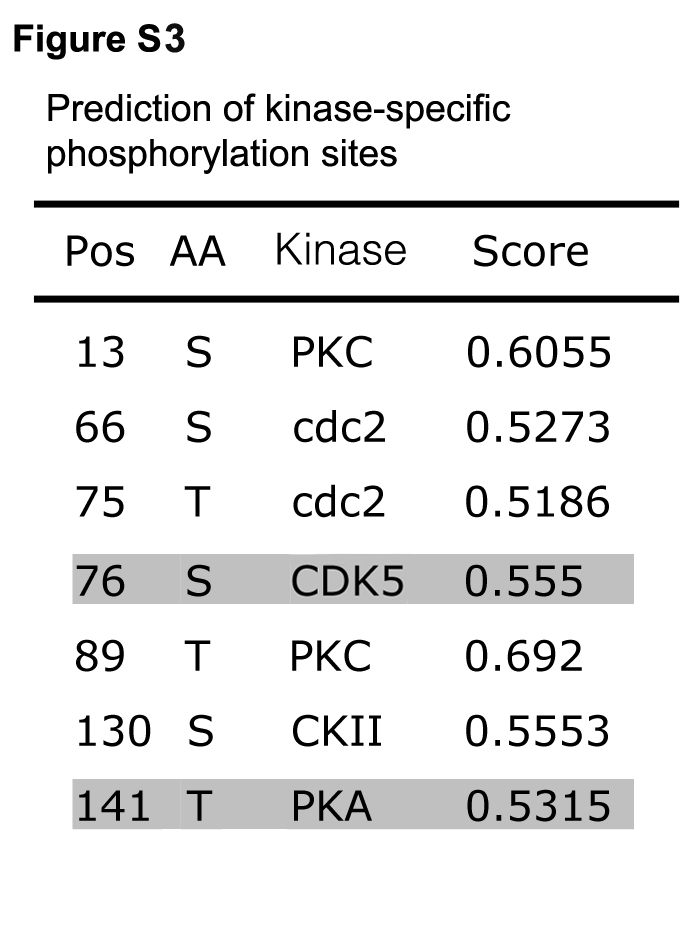

Supplement: Figure S3 — Prediction of kinase specific phosphorylation sites in p19. p19 protein sequence was analyzed for the presence of kinase specific phosphorylation sites using the bioinformatic tool NetphosK 1.0 server with evolutionary stable sites filter (ESS filter). Table shows the position of the putative phosphorylation sites for the indicated kinases. (Pos, position in p19 protein sequence). (TIF) [file pone.0035638.s004.tif]

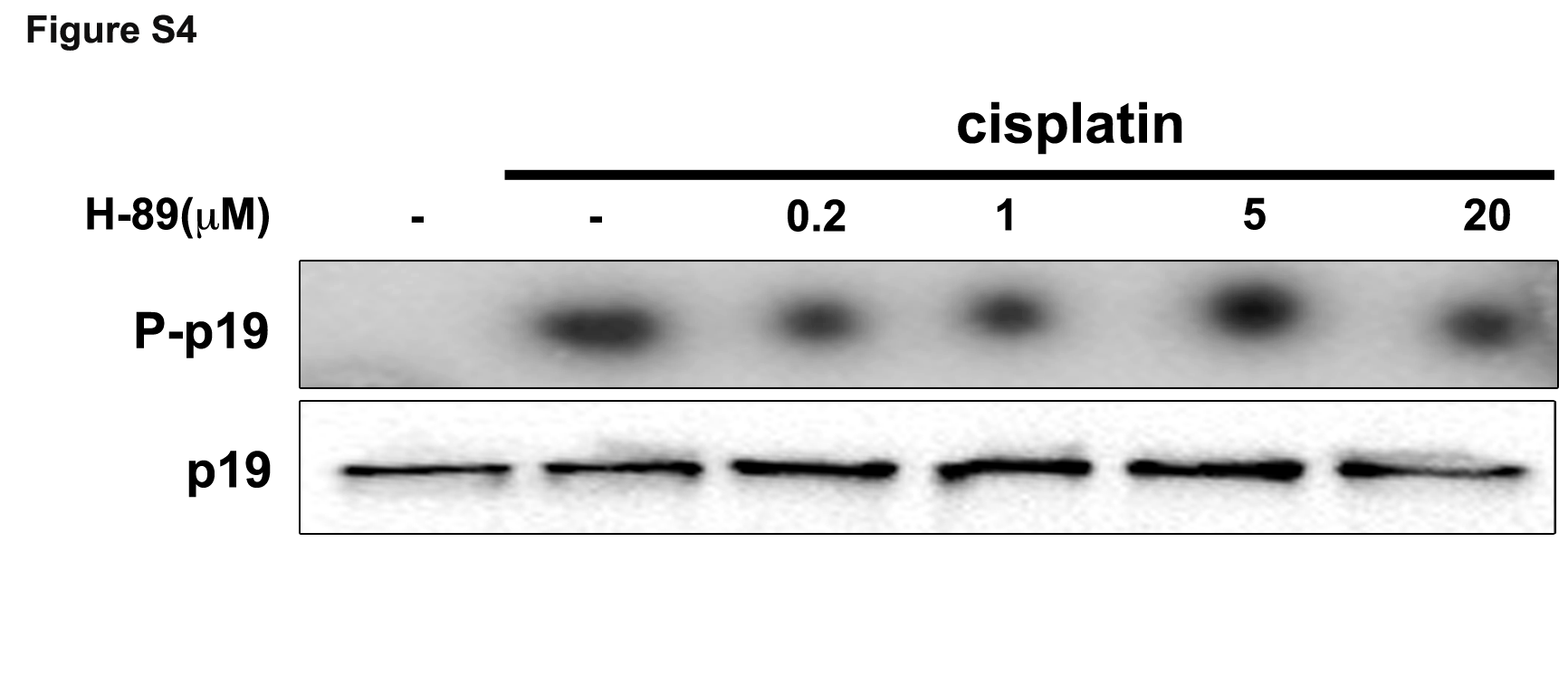

Supplement: Figure S4 — p19 phosphorylation is not abolished by high concentrations of PKA inhibitor. WI-38 fibroblasts were incubated with the indicated concentrations of H-89 for 1 hour, and then treated with cisplatin (10 µM) for 2 hours and endogenous p19 phosphorylation analyzed by autoradiography. (TIF) [file pone.0035638.s005.tif]

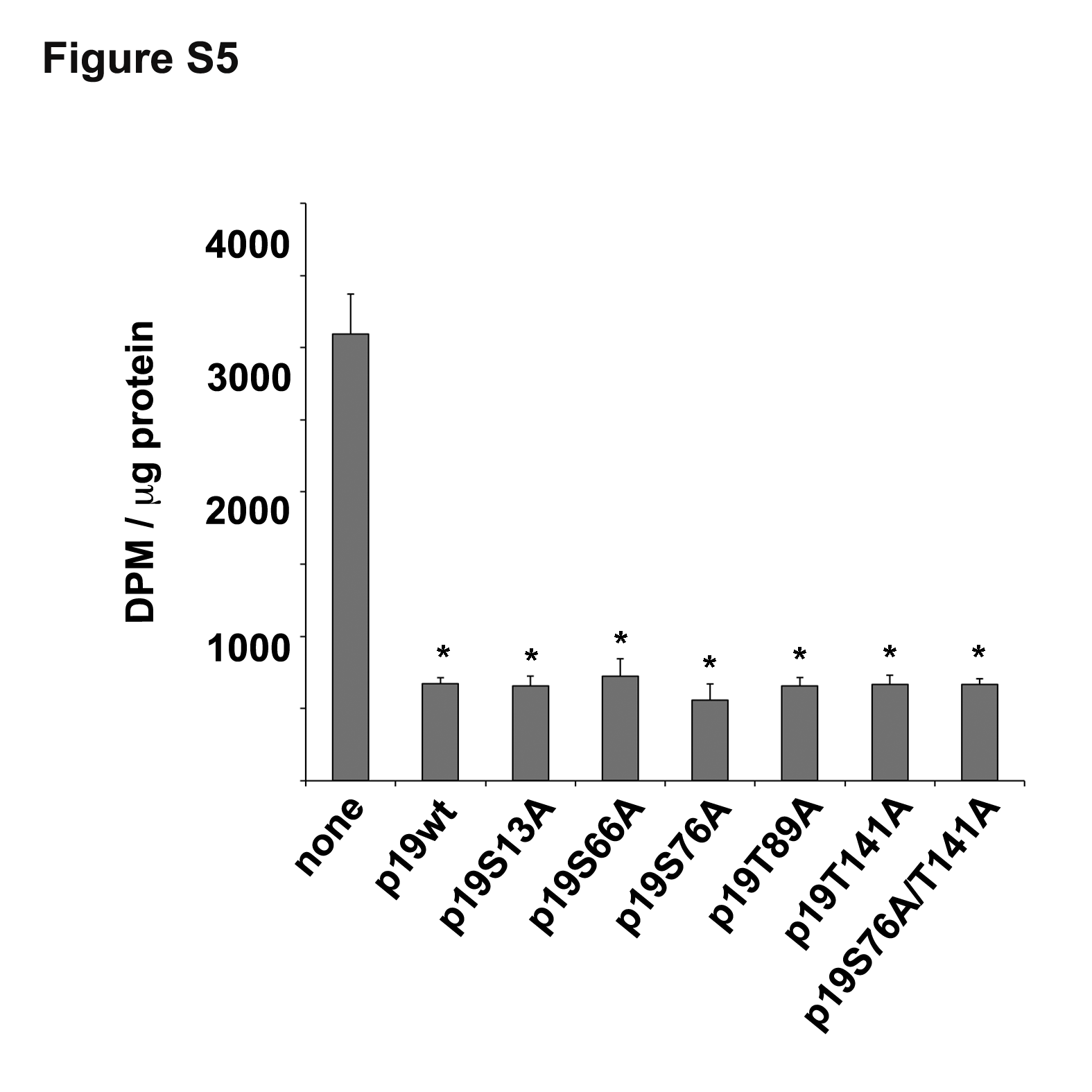

Supplement: Figure S5 — S76 and T141 are not involved in the cell cycle function of p19. Proliferation status of cells overexpressing p19wt or p19 phosphorylation deficient mutants. WI-38 fibroblasts were transfected with p19wt or the indicated p19 mutants. Cells were incubated with [3H]-thymidine for 5 hours and the lysates were tested for tritium incorporation. Bars represent the mean ± s.e.m of three independent experiments performed in triplicate. Student's t-test was used to compare control sample (none) with p19wt or p19 mutant samples. (*p<0,005). (TIF) [file pone.0035638.s006.tif]

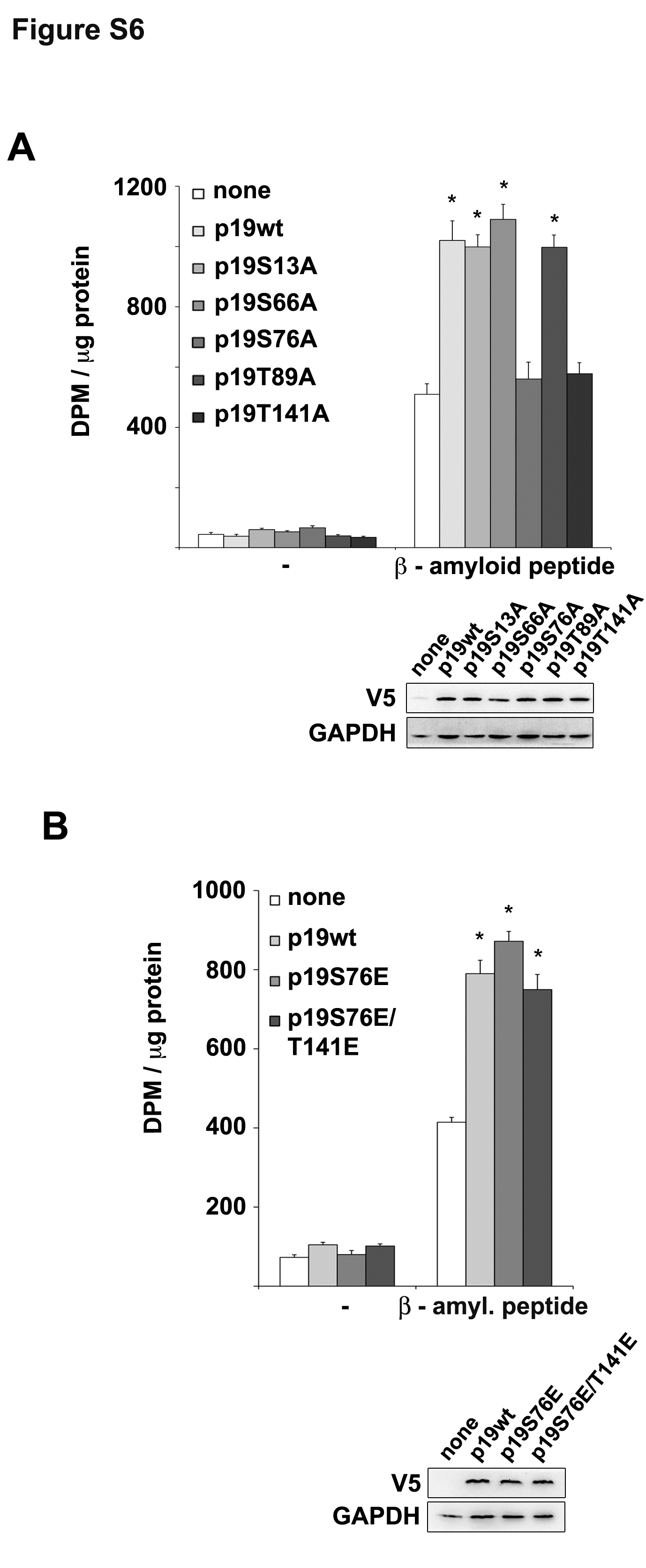

Supplement: Figure S6 — Phosphorylation of S76 and T141 is required for p19 function in DNA repair. (A) DNA repair ability of cells overexpressing p19wt or p19 phosphorylation deficient mutants. WI-38 fibroblasts were transfected with p19wt or the indicated p19 mutants. Cells were maintained in an arginine-free medium containing 1% fetal bovine serum during 48 h. β-amyloid peptide (20 µM) was added to the medium and cells were incubated with [3H]-thymidine for 10 hours. Cell lysates were tested for Unscheduled DNA Synthesis assay (UDS). Bars represent the mean ± s.e.m of three independent experiments performed in triplicate. Student's t-test was used to compare β-amyloid peptide-treated control sample (none) with β-amyloid peptide-treated p19wt or p19 mutant samples. (*p<0,005). Protein expression was analyzed by immunoblot. (B) Similarly as in (A) but overexpressing the phosphomimetic p19 mutants. (TIF) [file pone.0035638.s007.tif]

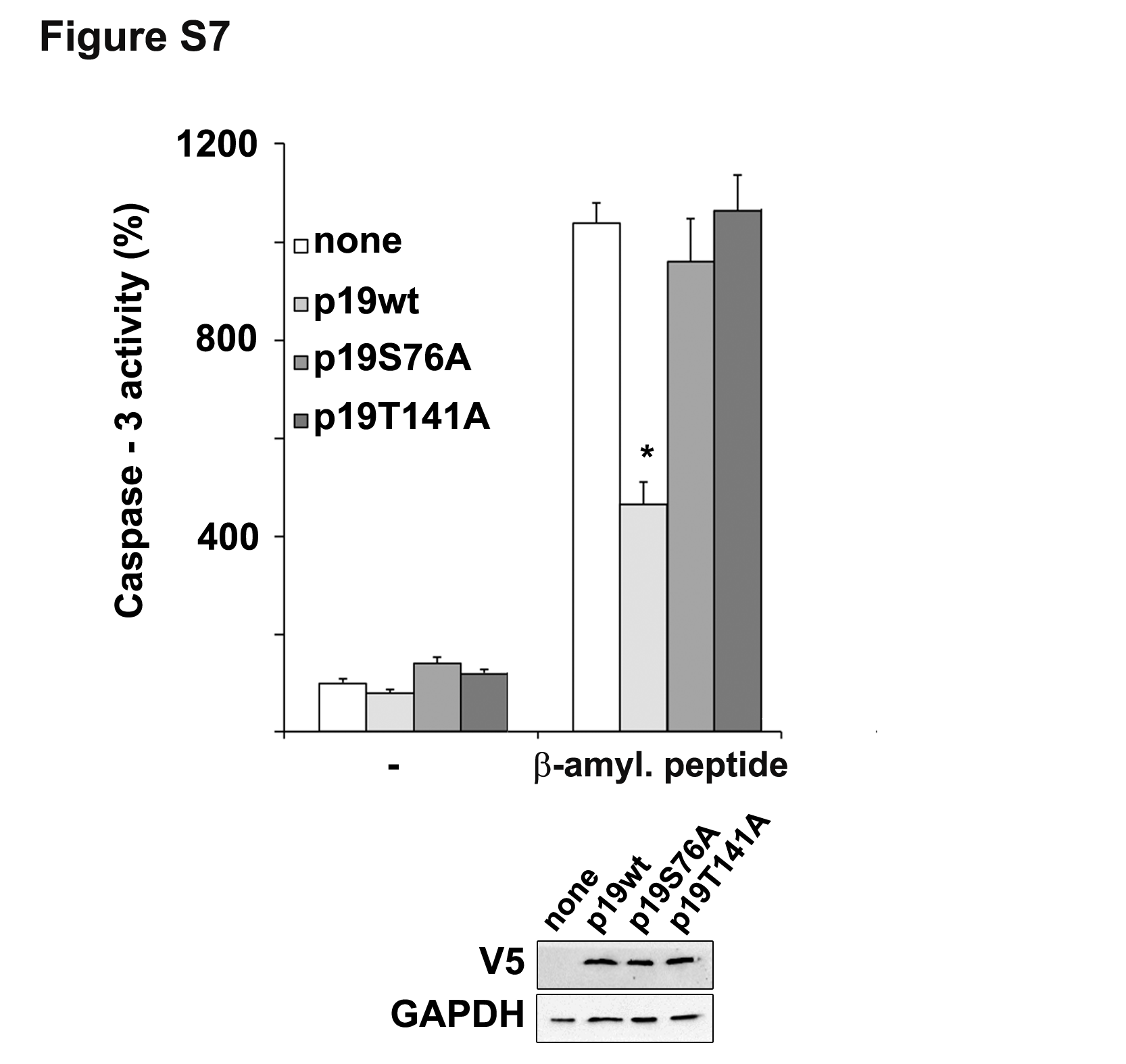

Supplement: Figure S7 — Phosphorylation of S76 and T141 is necessary for p19 function in apoptosis. β-amyloid peptide-dependent apoptotic response of cells overexpressing p19wt or the phosphorylation deficient mutants, p19S76A and p19T141A. WI-38 fibroblasts were transfected with p19wt or the indicated p19 mutants. β-amyloid peptide (20 µM) was added to the medium and following 12 hours cell lysates were tested for caspase-3 activity. Results are expressed as percentage of caspase-3 activity with respect to basal activity of cell lysates nontransfected and without β-amyloid peptide-treatment, which was set to 100. Bars represent the mean ± s.e.m of three independent experiments performed in triplicate. Students t-test was used to compare, β-amyloid peptide-treated control sample (none) with β-amyloid peptide-treated p19wt or p19 mutant samples (* p<0.005). (TIF) [file pone.0035638.s008.tif]

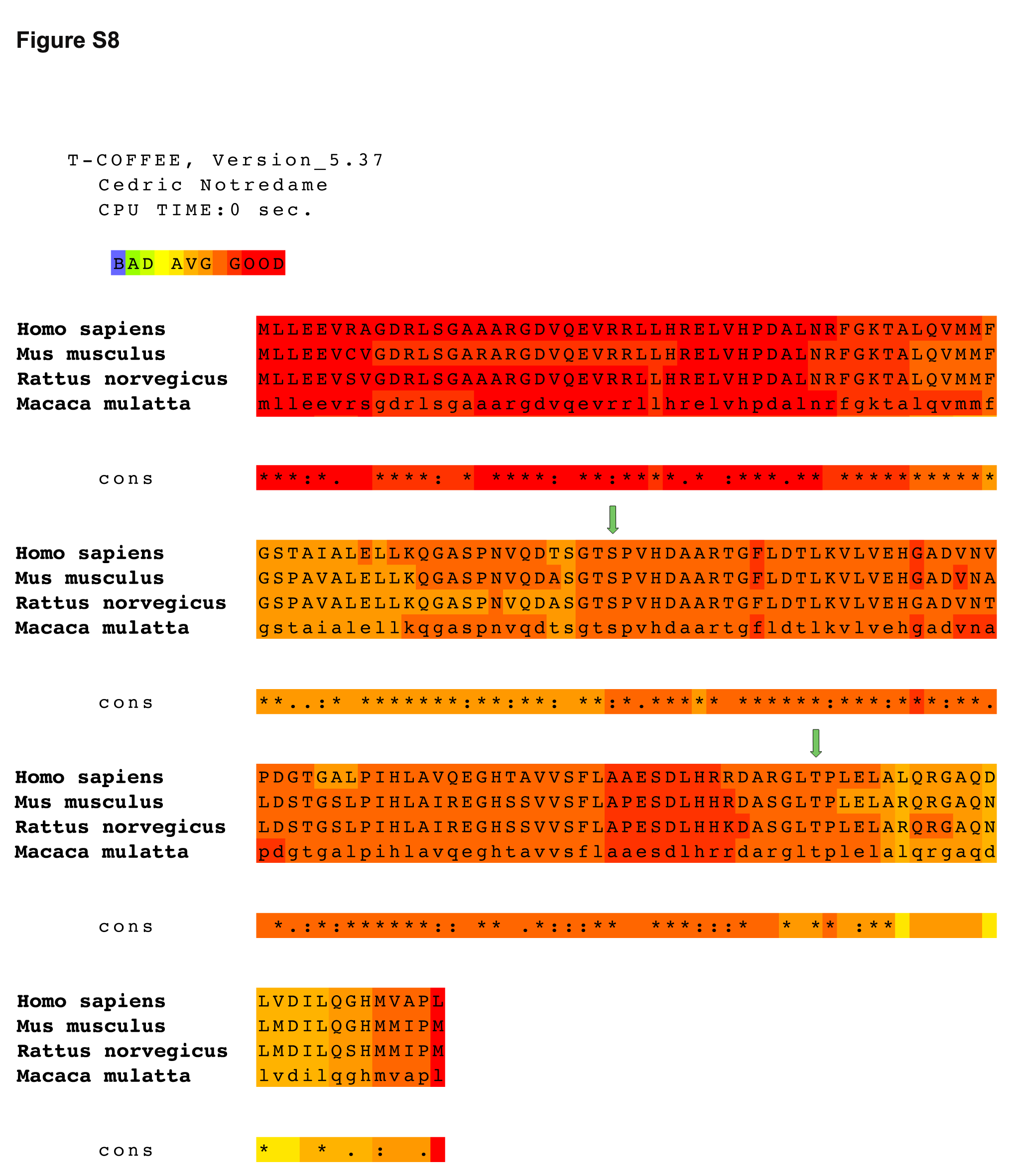

Supplement: Figure S8 — Conservation of p19 phosphorylation sites in different mammalian species. p19 protein sequences from the indicated mammals were align using T-Coffee multiple sequence alignment tool. Arrows indicate the position of S76 and T141 from p19 human sequence. (TIF) [file pone.0035638.s009.tif]

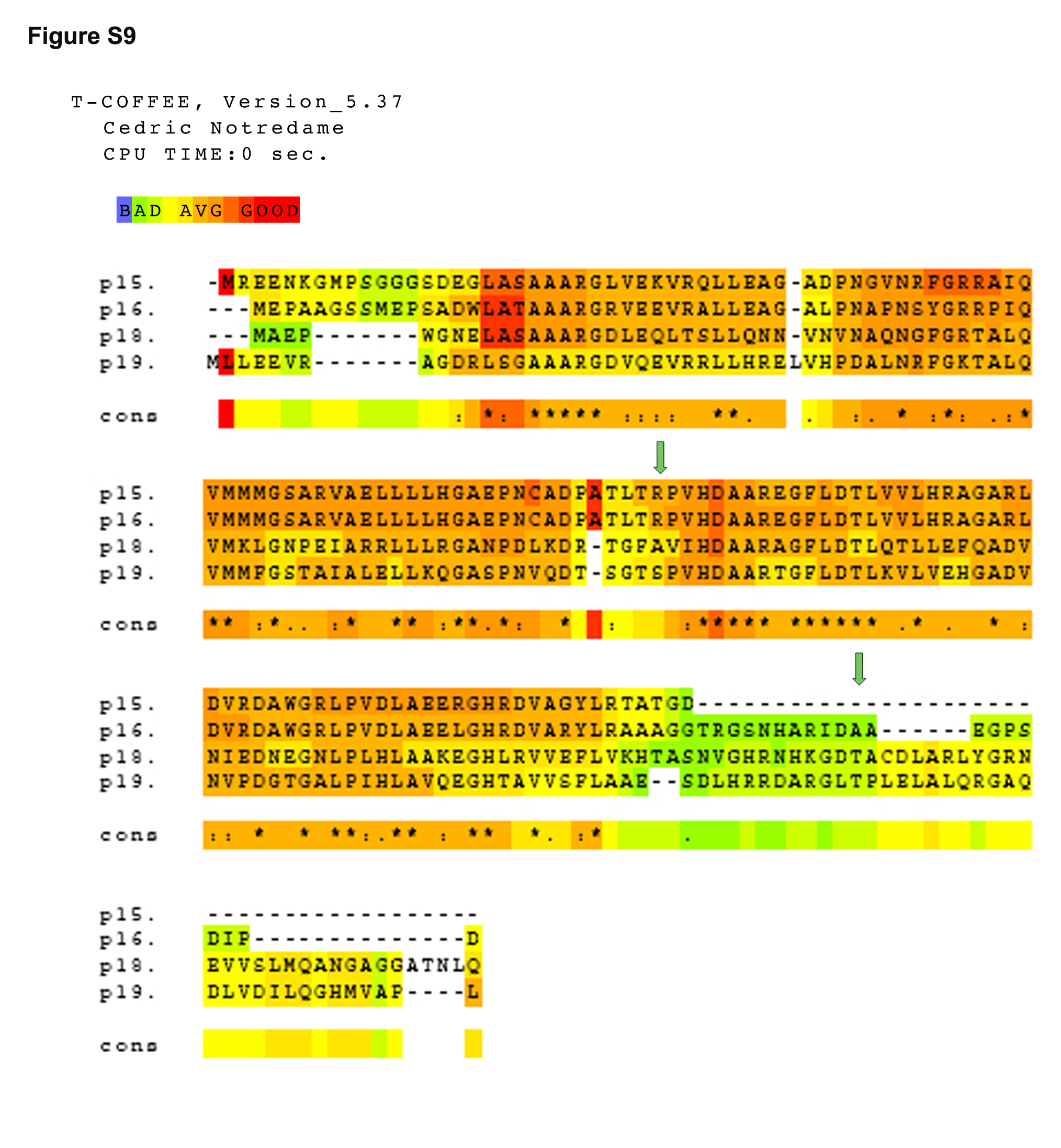

Supplement: Figure S9 — Alignment of protein sequences of the INK4 family members. Protein sequences were align using T-Coffee multiple sequence alignment tool. Arrows indicate the position of S76 and T141 from p19 protein sequence. (p15, p15INK4b; p16, p16INK4a; p18, p18INK4c; p19, p19INK4d). (TIF) [file pone.0035638.s010.tif]
